# Keeping up with the pathogens: improved antimicrobial resistance detection and prediction from *Pseudomonas aeruginosa* genomes

**DOI:** 10.1186/s13073-024-01346-z

**Published:** 2024-06-07

**Authors:** Danielle E. Madden, Timothy Baird, Scott C. Bell, Kate L. McCarthy, Erin P. Price, Derek S. Sarovich

**Affiliations:** 1 Centre for Bioinnovation, University of the Sunshine Coast, Sippy Downs, QLD Australia; 2 Sunshine Coast Health Institute, Birtinya, Queensland Australia; 3 Respiratory Department, Sunshine Coast University Hospital, Birtinya, Queensland Australia; 4 Adult Cystic Fibrosis Centre, The Prince Charles Hospital, Chermside, Queensland Australia; 5 Children’s Health Research Centre, Faculty of Medicine, The University of Queensland, South Brisbane, Queensland Australia; 6 University of Queensland Medical School, Herston, QLD Australia; 7 Royal Brisbane and Women’s Hospital, Herston, Queensland Australia

**Keywords:** Antibiotic, Genomics, High-throughput sequencing, Bioinformatics, AMR database, Metagenomics, Whole-genome sequencing, AMR software, AMR prediction

## Abstract

**Background:**

Antimicrobial resistance (AMR) is an intensifying threat that requires urgent mitigation to avoid a post-antibiotic era. *Pseudomonas aeruginosa* represents one of the greatest AMR concerns due to increasing multi- and pan-drug resistance rates. Shotgun sequencing is gaining traction for in silico AMR profiling due to its unambiguity and transferability; however, accurate and comprehensive AMR prediction from *P. aeruginosa* genomes remains an unsolved problem.

**Methods:**

We first curated the most comprehensive database yet of known *P. aeruginosa* AMR variants. Next, we performed comparative genomics and microbial genome-wide association study analysis across a Global isolate Dataset (*n* = 1877) with paired antimicrobial phenotype and genomic data to identify novel AMR variants. Finally, the performance of our *P. aeruginosa* AMR database, implemented in our AMR detection and prediction tool, ARDaP, was compared with three previously published in silico AMR gene detection or phenotype prediction tools—abritAMR, AMRFinderPlus, ResFinder—across both the Global Dataset and an analysis-naïve Validation Dataset (*n* = 102).

**Results:**

Our AMR database comprises 3639 mobile AMR genes and 728 chromosomal variants, including 75 previously unreported chromosomal AMR variants, 10 variants associated with unusual antimicrobial susceptibility, and 281 chromosomal variants that we show are unlikely to confer AMR. Our pipeline achieved a genotype-phenotype balanced accuracy (bACC) of 85% and 81% across 10 clinically relevant antibiotics when tested against the Global and Validation Datasets, respectively, vs. just 56% and 54% with abritAMR, 58% and 54% with AMRFinderPlus, and 60% and 53% with ResFinder. ARDaP’s superior performance was predominantly due to the inclusion of chromosomal AMR variants, which are generally not identified with most AMR identification tools.

**Conclusions:**

Our ARDaP software and associated AMR variant database provides an accurate tool for predicting AMR phenotypes in *P. aeruginosa*, far surpassing the performance of current tools. Implementation of ARDaP for routine AMR prediction from *P. aeruginosa* genomes and metagenomes will improve AMR identification, addressing a critical facet in combatting this treatment-refractory pathogen. However, knowledge gaps remain in our understanding of the *P. aeruginosa* resistome, particularly the basis of colistin AMR.

**Supplementary Information:**

The online version contains supplementary material available at 10.1186/s13073-024-01346-z.

## Background

Antibiotic overuse and misuse [1] has driven the emergence of antimicrobial-resistant (AMR) pathogens globally [2]. We are now on the verge of a ‘post-antibiotic era’, where simple infections threaten to be untreatable with antimicrobials that once revolutionised modern medicine [3]. If unmitigated, AMR infections are predicted to cause 10 million deaths globally by 2050 and cost USD$100 trillion per annum [4].

The ESKAPE pathogen, *Pseudomonas aeruginosa*, represents one of the biggest AMR threats due to its intrinsic resistance towards many antibiotics, environmental ubiquity, ability to infect a wide spectrum of hosts, and high global mortality rate [5–7]. Accurately detecting and predicting AMR phenotype from genotype in *P. aeruginosa* has proven challenging [8], even using machine learning [9], with some approaches as accurate as a coin flip [8]. A major shortcoming of current in silico AMR tools is that they largely focus on detecting AMR gene gain [10, 11] and a small number of chromosomally encoded single-nucleotide polymorphisms (SNPs) [12–14]. However, *P. aeruginosa* can also evolve AMR through chromosomal insertions-deletions (indels), loss-of-function mutations (e.g. large deletions or frameshift mutations), structural variants, and copy-number variations (CNVs) [15]. Despite recent advances [11, 12], most AMR tools remain limited in their scope and accuracy [16]—for example, loss-of-function mutations, a major contributor to AMR, are largely ignored [14], AMR databases are often not species-specific [14, 17], they do not resolve to the individual antibiotic level [11], and precursor mutations conferring reduced antimicrobial susceptibility are overlooked. These limitations are especially problematic for accurate AMR detection and prediction in pathogens encoding complex resistomes like *P. aeruginosa* [8].

To address this gap, we curated and validated the most comprehensive *P. aeruginosa*-specific AMR variant database yet, which, when used in conjunction with the Antimicrobial Resistance Detection and Prediction (ARDaP) software [14], enables high-accuracy AMR prediction from *P. aeruginosa* genomes. Performance of our ARDaP-compatible AMR variant database was first assessed across 1877 diverse *P. aeruginosa* strains (‘Global Dataset’) and, subsequently, across 102 analysis-naïve *P. aeruginosa* strains (‘Validation Dataset’). Our approach, which we demonstrate far exceeds the performance to current AMR prediction software, provides a crucial steppingstone towards the routine clinical use of genomics and metagenomics to inform personalised *P. aeruginosa* antimicrobial treatment regimens.

## Methods

### *P. aeruginosa* AMR variant database construction

To cover the spectrum of AMR variants found in *P. aeruginosa*, an ARDaP v2.3 [14] compatible SQLite database was populated with all known AMR variants described in this pathogen to date (https://github.com/dsarov/ARDaP/tree/master/Databases/Pseudomonas_aeruginosa_pao1/) [18]. Gene names, locus tags, and genomic coordinates in our AMR variant database were based on PAO1 (GCF_000006765.1) [19]. An exhaustive literature search was conducted to encompass biomedical literature published from 1980 to April 20, 2023, within the MEDLINE database. Search terms included ‘antimicrobial resistance’ and ‘*P. aeruginosa*’ accompanied by a variable search term for targeted literature, and included either (i) antibiotic class (e.g. carbapenem, aminoglycoside), (ii) AMR mechanism (e.g. efflux, gene expression enzymatic inactivation), or (iii) a known AMR gene (e.g. *oprD*, *ampC*, *gyrA*).

The resultant *P. aeruginosa* AMR database (Dataset 1) consists of three tables. The first table, ‘1. Antibiotics’, lists the ten clinically relevant antibiotics that were interrogated in our study: amikacin, cefepime, ceftazidime, ciprofloxacin, colistin, imipenem, meropenem, piperacillin, piperacillin/tazobactam, and tobramycin, whether these drugs are first- or second-line, and their associated antibiotic class. The second (‘2. AMR-conferring SNPs & indels’) and third (‘3. AMR gene coverage’) tables list the entire *P. aeruginosa* mutational resistome [20, 21], which includes all known genetic alterations that can lead to AMR, including the functional loss of chromosomal genes. These databases include all AMR variants and genes that (i) cause efflux pump upregulation [22], (ii) alter outer membrane permeability [23], (iii) de-repress or alter the substrate range of the AmpC cephalosporinase [24], and/or (iv) alter the antimicrobial target [15] . AMR gene acquisition was interrogated using the default parameters of ResFinder v4.0 [10], which is integrated into the ARDaP tool. To reduce false-positive and false-negative hits, the default ResFinder database was further curated to [1] remove loci (*bla*_OXA-395_1_AY306133_, *bla*_OXA-396_1_AY306134_, *bla*_PAO_4_AY083592_, *bla*_PAO_1_AY083595_, *bla*_PAO_3_FJ666073_, *bla*_PAO_2_FJ666065_, and *crpP*_HM560971) that were consistently identified in antimicrobial-sensitive strains; [2] include additional gene variants identified within the Global Dataset in aminoglycoside AMR-conferring genes *aac*(6’)-Ib, *aac*(6’)-Iia, *aac*(6’)-Ib-cr, and *aac*[3]-IIIa; and [3] expand the substrate range for *bla*_OXA-2_1_DQ112222_ and *bla*_OXA-2_2_GQ466184_ to include meropenem and imipenem, as per ResFinder recommendations for *P. aeruginosa*. Furthermore, only AMR genes with 100% similarity and 100% coverage are retained by ARDaP.

For the aminoglycosides (i.e. amikacin and tobramycin), known AMR variants consisted of SNPs in *algA* [25], *amgS* [26], *fusA1* [25, 27–32], *rplB* [9], *ptsP* [32, 33], and *tuf1* [25] and loss-of-function mutations in the *nuo* [34–36] pathway. For the carbapenems (i.e. imipenem and meropenem), cephalosporins (i.e. cefepime and ceftazidime), and β-lactams with or without an inhibitor (i.e. piperacillin and piperacillin/tazobactam), mutations that alter or expand the substrate range of the *ampC* cephalosporinase [24, 37–40] or that alter *ampC* expression, including inactivation of penicillin-binding-protein 4 (PBP4; *dacB*) [41–43], *ampD* [43–45], *ampDh2* [45], *ampDh3* [45], *ampE* [45], *ampR* [46, 47], *mpl* [48], or *ampC-ampR* intergenic region [49], mutations in PBP3 (*ftsI*) [46, 50, 51], and mutations that cause *oprD* loss, inactivation, or down-regulation [47, 52–58], were included. For ciprofloxacin AMR prediction, we included SNPs in *gyrA* [59–65], *gyrB* [60, 61, 63, 66], *parC* [59–61, 64], and *parE* [60, 61, 63]. For colistin AMR prediction, point mutations in *cprS* [50], *pmrA* [67], *pmrB* [30, 67–72], *phoP* [67], and *phoQ* [67] were included.

Efflux pumps play a key role in AMR development in *P. aeruginosa*, predominantly through regulator alterations, which drive efflux pump overexpression [15]. To predict MexAB-OprM upregulation, which is associated with β-lactam (including carbapenem) AMR [22], we included mutations in the *cis* (*mexR* [28, 59, 62, 73, 74]) and *trans* (*nalC* [75, 76] and *nalD* [24, 69, 75–77]) regulators of this efflux pump. For MexCD-OprJ upregulation, which is associated with fluoroquinolone and specific β-lactam AMR [22], mutations and loss-of coverage in the single known regulator, *nfxB* [59, 62] were included. For MexEF-OprN upregulation, which is linked to fluoroquinolone AMR [22], we included function-altering mutations or loss of coverage in *mexS* [78], the LysR family regulator *mexT* [79], and the global regulator *mvaT* [80]. For MexXY upregulation, which is associated with aminoglycoside and fluoroquinolone AMR, we included loss-of-coverage in two refined regions of *mexZ* [81, 82] and the intergenic region (*mexOZ*) between *mexZ* and *mexX* [83, 84]. Additionally, functional gene loss variants across the entire *P. aeruginosa* chromosome, identified via transposon insertion experiments [34, 36, 85], were included.

In addition to variants that confer AMR, mutations in essential AMR-conferring genes that cause unusual antimicrobial susceptibility were included: efflux pump loci *mexAB* [86, 87], *mexXY* [88], *mexC* [89] and *mexE* [80, 90], and the *ampC* regulators *ampP* and *ampG*, which are required for high-level *ampC* expression [49]. We also included additional targets for (meta)genome quality control (*ecfX* [91]) for *P. aeruginosa* speciation and genes responsible for conferring a hypermutator phenotype (*mutS*, *mutL*, and *uvrD* [92]).

### Global Dataset

To comprehensively capture geographic, genomic, and phenotypic diversity, we collated a large and comprehensive *P. aeruginosa* isolate collection (*n* = 1877) of all publicly available strains with paired antimicrobial phenotype and genomic data [9, 21, 28, 31, 50, 52, 93–99] (Table 1, Dataset 2, Table S1). This dataset includes many antimicrobial-susceptible strains (Table 1), which are essential for developing and refining high-quality AMR databases [14, 100]. Isolates with minimum inhibitory concentration (MIC) data were reclassified as sensitive, intermediate, or resistant using the CLSI M100S-Ed32:2022 guidelines.

**Table 1:** Summary of antimicrobial resistance prevalence across 10 clinically relevant antibiotics in the *Pseudomonas aeruginosa* Global Dataset

**Antibiotic class**	**Antibiotic**	**Res (%)**	**Int (%)**	**Sen (%)**	**No. isolates**
Carbapenems	MEM	682 (36)	149 (8)	682 (36)	1875
	IPM	186 (29)	46 (7)	419 (64)	651
Polymyxins	CST	48 (5)	0 (0)	933 (95)	981
Fluoroquinolones	CIP	664 (54)	78 (6)	477 (39)	1219
Cephalosporins	FEP	157 (17)	111(12)	650 (71)	918
	CAZ	316 (27)	121 (10)	733 (63)	1170
Penicillins	TZP	179 (24)	52 (7)	526 (69)	757
	PIP	185 (36)	55 (11)	272 (53)	512
Aminoglycosides	TOB	294 (29)	12 (1)	695 (69)	1001
	AMK	170 (12)	56 (4)	1199 (84)	1425

*Abbreviations*: *Res* resistant, *Int* intermediate, *Sen* sensitive, *MEM* meropenem, *IPM* imipenem, *CST* colistin, *CIP* ciprofloxacin, *FEP* cefepime, *CAZ* ceftazidime, *TZP* piperacillin/tazobactam, *PIP* piperacillin, *TOB* tobramycin, *AMK* amikacin

### Validation Dataset

To independently evaluate AMR software performance, we examined 102 phylogenetically diverse, analysis-naïve, clinical *P. aeruginosa* strains (Fig. S1 and Fig. S2). Isolates were obtained from people admitted to hospitals in Qld, Australia, with cystic fibrosis (CF; *n* = 42), bacteraemia (*n* = 35), chronic obstructive pulmonary disease (COPD; *n* = 21), bronchiectasis (*n* = 1), ear infection (*n* = 1), ulcer (*n* = 1), or urinary tract infection (*n* = 1), between 2008 and 2020 (Table S2). Eighty-four isolates have previously been genome-sequenced (NCBI BioProject PRJNA761496 [101]; GenBank accessions NSXK00000000.1 and NSZK00000000.1 [102]), and some have previously undergone antimicrobial susceptibility testing [101, 103]. For the current study, 18 additional COPD isolates (SCHI0038.S.1, SCHI0050.S.3, SCHI0058.S.1, SCHI0058.S.2, SCHI0059.S.1, SCHI0064.S.1, SCHI0065.S.1, SCHI0068.S.3, SCHI0070.S.1, SCHI0070.S.1, SCHI0084.S.1, SCHI0098.S.1, SCHI0103.S.1, SCHI0107.S.1, SCHI0109.S.1, SCHI0109.S.2, SCHI0112.S.1, SCHI0112.S.2) were sequenced and appended to BioProject PRJNA761496. Antimicrobial susceptibility profiles were determined across the Validation Dataset (Table S2) using disc diffusions for 8 of 10 clinically relevant antibiotics, following CLSI M100S-Ed32:2022 guidelines, whereas meropenem and ciprofloxacin MICs were determined by ETEST (bioMérieux, Murarrie, Australia) using sensitive, intermediate, and resistant MIC cut-offs of ≤ 4, 8, and ≥ 16 μg/mL for meropenem and ≤ 0.5, 1, and ≥ 2 μg/mL for ciprofloxacin. PAO1 (Belgian Coordinated Collection of Microorganisms [BCCM], Ghent University, Belgium) and LMG 6395 (BCCM) were included as antimicrobial-susceptible controls.

### Microbial genome-wide association study (mGWAS) and machine learning for AMR prediction

To identify novel AMR variants, mGWAS [104] was performed on the Global *P. aeruginosa* Dataset (*n* = 1877 strains), with SNPs and indels identified using SPANDx v4.0.1 [105]. To increase the signal-to-noise ratio, variants found in antimicrobial-sensitive isolates were penalised four-fold compared with AMR strains due to a presumed large effect size [106]. The top 50 variants associated with each AMR phenotype were assessed for their ability to improve phenotype prediction; those that improved phenotype prediction were included in the AMR database. Additionally, a supervised machine learning approach was performed using the Global Dataset and the AMR database as features for model creation.

### Comparative genomic analysis

To identify additional novel AMR variants, we conducted a comparative genomic analysis using SPANDx, with a focus on AMR strains that did not encode a known AMR variant (i.e. false negatives). These strains were compared to their closest antimicrobial-sensitive relative(s) as determined by the whole-genome phylogenetic analysis (Fig. S1). SNPs and indels that separated AMR from antimicrobial-sensitive strain/s were identified, annotated, and manually investigated to prioritise mutations in known AMR genes. Candidate variants were then tested against the Global Dataset to determine whether they improved phenotype prediction. AMR variants that increased balanced accuracy (bACC) were included in the database; those that did not alter, or that decreased bACC, were discarded.

### AMR prediction analysis

AMR prediction was performed using our *P. aeruginosa* AMR database (version 1.0), implemented in ARDaP [14]. ARDaP was chosen as it is the only AMR software that can detect all mutation types (i.e. SNPs, indels, gene gain, gene loss, frameshift mutations, structural variants, and CNVs) [14]. ARDaP also has a built-in feature that automatically generates a clinician-friendly antimicrobial susceptibility summary report for each strain (Fig. S3) to simplify in silico AMR interpretation [14]. ARDaP performance was compared against four tools for AMR phenotype prediction and/or AMR variant identification: abritAMR [107], RGI v5.1.0 and CARD v3.0.9 [11], ResFinder v4.1 [10], and AMRFinderPlus v3.8.28 [108]. As abritAMR and AMRFinderPlus frequently report predicted AMR phenotypes to the antibiotic class level only, we chose to interpret AMR variant presence for a given class as conferring AMR towards all antibiotics within that class. Importantly, AMRFinderPlus is not intended for clinical phenotype prediction [109] but has been included as a benchmark for gene detection accuracy. For the purposes of software comparisons, gene identification by AMRFinderPlus was interpreted as conferring phenotypic AMR.

A variant scoring scheme has previously been described by Cortes-Lara and colleagues, which employed a 0 (no effect) through 1 (EUCAST AMR) scale to predict in silico AMR profiles [98]. We expanded upon this scheme by providing an automated weighted score for all AMR variants in our database that quantifies their contribution, positive or negative, towards AMR development (Dataset 1, ‘Threshold’ column); this score is recorded for each antibiotic on ARDaP’s automatically generated clinician-friendly report (Fig. S3), unlike the Cortes-Lara scheme, which requires manual scoring for each antibiotic and strain [98]. Using our scoring system, variants known to cause AMR in isolation score as 100%, whereas AMR variants known to confer AMR in a stepwise manner (that is, only when in combination with other variant/s), or that only result in intermediate resistance, are given a lower score (e.g. 40–50%). This method accounts for both the additive nature of chromosomal mutations in *P. aeruginosa* and for the decreased AMR potential caused by loss of efflux pumps or essential transcriptional regulators. For the purposes of phenotype prediction, acquired AMR genes identified by ResFinder within ARDaP were considered to confer full AMR, i.e. given a threshold score of 100.

#### Intermediate resistance prediction

The capacity of our AMR variant database to predict intermediate resistance phenotypes was examined against the Global Dataset using the following criteria:

- A true-positive prediction occurred when either (i) an AMR strain was classed as AMR or (ii) an intermediate strain was identified as intermediate;
- A true-negative prediction occurred when an antimicrobial-sensitive strain was identified as antimicrobial sensitive;
- A false-positive prediction occurred when either (i) an antimicrobial-sensitive strain was classed as intermediate or AMR or (ii) an intermediate strain was classed as AMR; and
- A false-negative prediction occurred when either (i) an intermediate strain was classed as antimicrobial-sensitive or when an AMR strain was predicted to be antimicrobial-sensitive or intermediate.

These rules provided the strictest evaluation criteria for the assessment of ARDaP’s ability to identify intermediate resistance strains. Only ARDaP’s intermediate resistance prediction performance was assessed as abritAMR, AMRFinderPlus, CARD, and ResFinder all lack the capacity to identify intermediate resistance.

#### *Pseudomonas*-derived cephalosporinase (bla_PDC_) genotypes

Amino acid variants of the chromosomally encoded *P. aeruginosa ampC* β-lactamase gene are synonymously referred to as *bla*_PDC_ variants in some literature [110] [36], including in CARD outputs [11]. To enable genotype correlations with the *bla*_PDC_ nomenclature scheme, we incorporated the 476 CARD-described *bla*_PDC_ variants into ARDaP’s ResFinder database. These genotypes are output by default into /Outputs/Resfinder for each isolate included in the analysis.

#### AMR software predictive performance in *P. aeruginosa*

Due to its inability to predict AMR towards individual antibiotics, and a very high rate of false-positive predictions in the Global Dataset, CARD was deemed unsuitable for *P. aeruginosa* AMR analysis and was thus excluded from further assessment. For all other tools, predictive performance was determined using bACC [111, 112], which averages sensitivity [i.e. true positives/(true positives + false negatives)] and specificity [i.e. true negatives/(true negatives + false positives)]. This metric was chosen as it accounts for dataset imbalance, that is, it minimises over- or under-representation of antimicrobial-sensitive or AMR strains that may otherwise make certain tools appear better or worse due to inherent dataset bias [8]. Additionally, we compared recall (AMR) [true positives/(true positives + false negatives], precision (AMR) or positive predictive value [true positives/(true positives + false positives), recall (sensitivity) [true negatives/(true negatives + false positives), and precision (sensitivity) or negative predictive value [true negatives/(true negatives + false negatives)] across all software tools.

## Results

### *P. aeruginosa* AMR variant identification and refinement

An extensive literature search was undertaken to identify all known and putative chromosomal variants that lead to AMR in *P. aeruginosa*. Among 643 previously reported chromosomal AMR variants in known AMR loci (Dataset 1), 362 (56.3%) were confirmed to be associated with AMR, whereas 281 (43.7%) were re-classified as ‘natural variation’ (Table S3) as they were common in both antimicrobial-sensitive and AMR strains in our Global Dataset and therefore deemed unlikely to contribute to an AMR phenotype. Most of these naturally occurring variants had been previously reported as putatively causing AMR, with little to no functional investigation. Importantly, no functionally validated AMR driving variants were re-classified as ‘natural variation’. The loss of a further 10 genes was associated with unusual antimicrobial sensitivity (Dataset 1). Next, using mGWAS and comparative genomic analyses of the Global Dataset, we identified 75 previously unreported AMR variants associated with one or more AMR phenotypes (Table 2). In total, our *P. aeruginosa* database contains 437 chromosomal AMR variants, 281 natural variants, and 10 genes associated with unusual sensitivity (Dataset 1), along with 3639 refined mobile AMR genes derived from ResFinder.

**Table 2:** Novel antimicrobial resistance (AMR) variants identified in *Pseudomonas aeruginosa* by microbial genome-wide association study (mGWAS) or comparative genomic^a^ analyses

**Locus**	**Type of mutation**	**Associated AMR phenotype**	**Fisher’s exact *p* value**
*PA0027*	Lys34Asn	PIPr	< 0.0001
*PA0091* (*vgrG1*)	Gln214His	AMKi	NA
*PA0746*	Lys106Asn	FEPr	< 0.0001
*PA0958* (*oprD*)	Thr18fs^a,c^	MEMr, IPMr	NA
*PA0958* (*oprD*)	Phe36fs^a,c^	MEMr, IPMr	NA
*PA0958* (*oprD*)	Leu136Gln^a^	MEMr	NA
*PA0958* (*oprD*)	Gly139fs^a,c^	MEMr, IPMr	NA
*PA0958* (*oprD*)	Gly193fs^a,c^	MEMr, IPMr	NA
*PA0958* (*oprD*)	Tyr199fs^a,c^	MEMr, IPMr	NA
*PA0958* (*oprD*)	Gln340Pro^a^	MEMr	NA
*PA0958* (*oprD*)	Tyr350fs^a,c^	MEMr, IPMr	NA
*PA0958* (*oprD*)	Leu355fs^a,c^	MEMr, IPMr	NA
*PA0958* (*oprD*)	Leu409Pro^a^	MEMr	NA
*PA1430* (*lasR*)	Glu183Gly^a^	CSTr	NA
*PA1456* (*cheY*)	Val81Ala	CSTr	NA
*PA1760*	LOF^a^	TZPi	NA
*PA1798* (*parS*)	Ala149Thr	MEMr	< 0.0001
*PA1798* (*parS*)	Arg383Cys^a^	CSTr	NA
*PA1798* (*parS*)	Arg7His^a^	PIPr	NA
*PA1798* (*parS*)	Asp249Asn^a^	TZPr, AMKi	NA
*PA1799* (*parR*)	Val68Ala	CAZr	0.0002
*PA1799* (*parR*)	Leu165Phe^a^	MEMr	NA
*PA1923*	Ala350Val	TZPr	< 0.0001
*PA1942*^1^	LOF^a^	MEMr, IPMr	NA
*PA2020 (mexZ)*	Thr32Asn^a^	MEMr	NA
*PA2020 (mexZ)*	Ser198Ile^a^	MEMr	NA
*PA2020* (*mexZ*)	Leu199Arg^a^	FEPr	NA
*PA2402*	Val3040Ala	AMKr	< 0.0001
*PA2520* (*czcA*)	Gly1051Asp^a^	CSTr	NA
*PA2736*	Arg30Gly	AMKr	< 0.0001
*PA3047* (*PBP4, dacB*)	Thr27Ser	CAZr, FEPr, PIPr, TZPr	NA
*PA3047* (*PBP4, dacB*)	Ser230Ile	CAZr, FEPr, PIPr, TZPr	NA
*PA3047* (*PBP4, dacB*)	Phe438Leu	CAZr, FEPr, PIPr, TZPr	NA
*PA3083* (*pepN*)	LOF^a^	MEMr, CAZr	NA
*PA3384* (*phnC*)	LOF^a^	MEMr, TOBr	NA
*PA3574* (*nalD*)	Leu76Pro^a^	MEMr	NA
*PA3574* (*nalD*)	Arg164Pro^a^	PIPr, FEPr	NA
*PA3721* (*nalC*)	Lys58Glu^a^	MEMr	NA
*PA4020* (*mpl*)	Gly137Ser^a^	CAZr	NA
*PA4109* (*ampR*)	Ter297Tyr stop lost^a,c^	PIPr	NA
*PA4110* (*ampC*)	Asp272Asn^a^	MEMr	NA
*PA4110* (*ampC*)^2^	Leu320Pro^a^	MEMr	NA
*PA4260* (*rplB*)	Gly138Ser^a^	TOBi, AMKr	NA
*PA4266* (*fusA1*)^c^	Ser459Phe^a^	TOBr, AMKr	NA
*PA4296* (*pprB*)	Asn253His^a^	CSTr	NA
*PA4318*	Pro243Ser	PIPr	< 0.0001
*PA4418* (*ftsI*)	Gly63Asp^a^	IPMr, CAZr, FEPr, TZPr, PIPr	NA
*PA4522* (*ampD*)	Val10Gly^a^	CAZr, FEPi, PIPi	NA
*PA4522* (*ampD*)	Pro41Leu^a^	PIPr, FEPr	NA
*PA4522* (*ampD*)	Glu67stop^a,c^	CAZi, MEMi, IPMi, TZPi, PIPi, FEPi	NA
*PA4522* (*ampD*)	Ile69Thr^a^	MEMr	NA
*PA4522* (*ampD*)	Gln88Leu^a^	CAZr	NA
*PA4522* (*ampD*)	Gly100Glu^a^	MEMr	NA
*PA4522* (*ampD*)	Cys110Gly^a^	FEPr	NA
*PA4522* (*ampD*)	Gly116Val^a^	MEMr, CAZr	NA
*PA4522* (*ampD*)	Ile117Thr^a^	CAZr	NA
*PA4522* (*ampD*)	Gly121Glu^a^	MEMr, CAZi	NA
*PA4522* (*ampD*)	His157Tyr^a^	CAZr	NA
*PA4522* (*ampD*)	Gly169Cys^a^	FEPr	NA
*PA4599* (*mexC*)	-58C>A^a^	FEPr	NA
*PA4677*	Asp138Val	FEPr	< 0.0001
*PA4776* (*pmrA*)	Leu72Phe^a^	CSTr	NA
*PA4777* (*pmrB*)	Arg10Leu^a^	CSTr	NA
*PA4777* (*pmrB*)	Arg155His^a^	CSTr	NA
*PA4777* (*pmrB*)	Thr158Ile^a^	CSTr	NA
*PA4777* (*pmrB*)	Thr253Met^a^	CSTr	NA
*PA5000* (*wapR*)	Gly163Asp^a^	CSTr	NA
*PA4967* (*parE*)	Glu459Lys	CIPi	NA
*PA4967* (*parE*)	Val520Ala	CIPi	NA
*PA5045* (*ponA*)	Met671Ile	CAZr, FEPr, TZPr, PIPr	NA
*PA5051* (*argS*)	Asp184Gly	TOBr	NA
*PA5199* (*amgS*)	Trp120Gly^a^	CSTr	NA
*PA5199* (*amgS*)	Pro435Ala^a^	CSTr	NA
*PA5338* (*spoT*)	Gly496Ser^a^	CAZr	NA
*PA5493* (*polA*)	Lys395Arg^a^	AMKr	NA

*Abbreviations*: *AMK *amikacin, *CAZ* ceftazidime, *CIP* ciprofloxacin, *CST* colistin, *FEP* cefepime, *fs* frameshift, *i* intermediate resistant, *IPM* imipenem, *LOF* loss of function, *MEM* meropenem, *NA* not applicable, *PIP* piperacillin, *r* resistant, *TOB* tobramycin, *TZP* piperacillin/tazobactam

^1^Previously predicted to cause PIPr [36]; however, our microbial genome-wide associate study (mGWAS) analysis did not identify a significant association with this phenotype. Instead, mGWAS showed that this AMR variant was significantly associated with MEMr and IPMr

^2^Previously identified variant in *ampC* known to reduce susceptibility to multiple cephalosporins

^a^Variant identified by comparative genomics and thus not assessed for statistical significance

^b^Identified in the Validation Dataset only

^c^High-consequence mutations occurring in this gene are automatically identified by ARDaP

### Predictive performance across Global Dataset

**Fig. 1:**
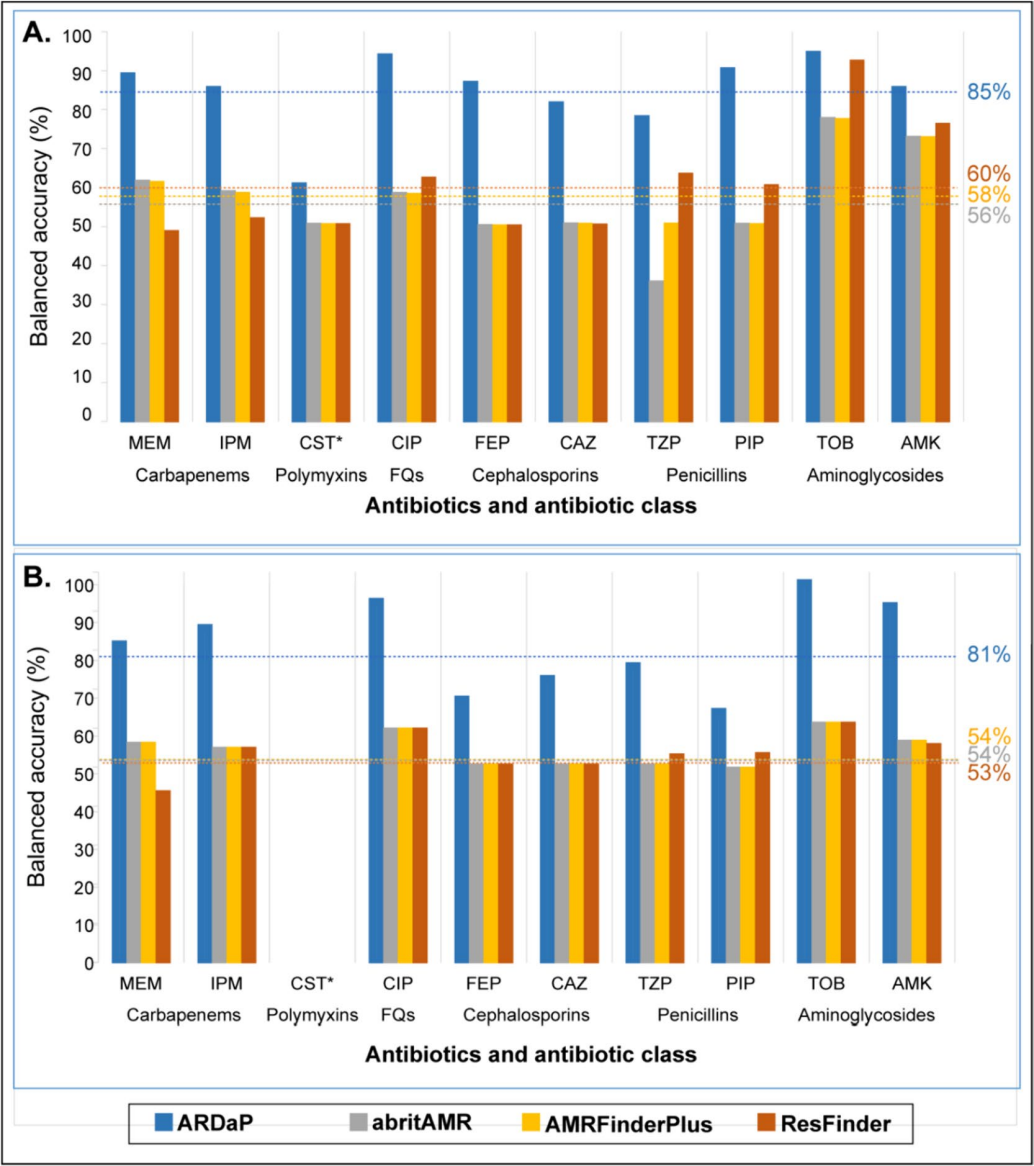
Balanced accuracy of ARDaP, abritAMR, AMRFinderPlus, and ResFinder software for antimicrobial resistance (AMR) prediction in *Pseudomonas aeruginosa*. Software comparisons across ten clinically relevant antibiotics were undertaken against **A** the Global Dataset (*n* = 1877 isolates) and **B** the Validation Dataset (*n* = 102 isolates). For both datasets, and for all 10 antibiotics, ARDaP outperformed abritAMR, AMRFinderPlus, and ResFinder. To enable comparison with existing AMR prediction software, isolates with intermediate resistance were removed prior to analysis. Abbreviations: AMK, amikacin; CAZ, ceftazidime; CIP, ciprofloxacin; CST, colistin; FEP, cefepime; FQs, fluoroquinolones; IPM, imipenem; MEM, meropenem; PIP, piperacillin; TZP, piperacillin/tazobactam; TOB, tobramycin. “*” symbol indicates the following: no strains in the Validation Dataset exhibited CST AMR; as such, balanced accuracy could not be calculated for this antibiotic

Although superior to CARD, abritAMR, and AMRFinderPlus, ResFinder still showed relatively poor bACCs for most antibiotics, with an average bACC of 60%, well below ARDaP’s average bACC of 85% (Fig. 1A).

The best abritAMR, AMRFinderPlus, and ResFinder predictions were achieved for the aminoglycosides, with average bACCs of 75%, 75%, and 84%, respectively, although these rates were lower than ARDaP’s average aminoglycoside bACC of 92%. abritAMR, AMRFinderPlus, and ResFinder AMR prediction for all other antibiotics showed poor to very poor bACCs. AMRFinderPlus had a bACC of just 50% for the penicillins, cephalosporins, and colistin—the same predictive capacity as a coin flip—and had only a slightly better bACC for ciprofloxacin (58%), imipenem (58%), and meropenem (61%). ResFinder also had a bACC of just 50% for cephalosporins and colistin, and performed worse for carbapenems (average bACC of 50%) than AMRFinderPlus, although it was better for penicillins (average bACC of 62%) and ciprofloxacin (bACC of 62%). abritAMR was the worst at predicting penicillin phenotypes, with an average bACC of just 43%, worse than a coin flip; its performance was otherwise identical to AMRFinderPlus. In contrast, ARDaP surpassed abritAMR, AMRFinderPlus, and ResFinder across all 10 antibiotics, with bACCs ranging from 60% (colistin) to 94% (tobramycin) (Fig. 1A).

We next assessed the role of natural variants on ARDaP’s AMR prediction performance. As expected, exclusion of natural variation resulted in a lower bACC for some antibiotics, most notably for the carbapenems, which dropped from 89% to 70%, and ciprofloxacin, which dropped from 93% to 56%. This loss of accuracy was caused by an increase in *oprD* and *mexT* false positives that were incorrectly predicted to confer carbapenem and ciprofloxacin AMR, respectively.

In comparison to other AMR prediction/gene identification tools, the increase in ARDaP’s predictive performance was predominantly due to accurate identification of chromosomal SNP and indel variants. For the carbapenems, this increase was due to the identification of loss-of-function mutations in *oprD*, with all tools successfully identifying other non-chromosomal variants (e.g. *bla*_VIM_). A similar trend was also observed for the other antibiotics. For example, the increase in CIP accuracy was due to ARDaP identifying *gyrA* mutations, which were not identified by other tools.

### Predictive performance across the Validation Dataset

We next tested abritAMR, AMRFinderPlus, ARDaP, and ResFinder across the Validation Dataset of 102 Australian clinical *P. aeruginosa* strains (Table S2) to determine each software’s performance in an analysis-naïve dataset. As no strains in the Validation Dataset displayed colistin AMR, the bACC for this antibiotic could not be assessed. These strains otherwise exhibited similar AMR rates to the Global Dataset, ranging from 26% for meropenem to 57% for piperacillin (Table S4).

Overall, ARDaP had high predictive accuracy (average bACC of 81%) across all antibiotics (Fig. 1B), outperforming ResFinder (average bACC of 53%), abritAMR, and AMRFinderPlus (average bACC of 54% each). Notably, the ResFinder meropenem bACC, at just 43%, yielded the poorest score.

### Inclusion of novel AMR variants identified in the Global Dataset

The inclusion of these markers increased Validation Dataset sensitivity by an average of 4% (range 0 to 27%) depending on antibiotic, with the sensitivity of most antibiotics (meropenem, imipenem, ciprofloxacin, cefepime, and piperacillin/tazobactam) remaining unchanged. Amikacin increased the most (27%) due to the inclusion of a SNP in *rplB* (Gly138Ser), followed by tobramycin at 4%, and piperacillin and ceftazidime at 3% each.

### ARDaP performance between the Global and Validation Datasets

Whilst ARDaP bACC between the datasets were broadly similar, there was a greater proportion of false-positive and false-negative variants encoding AMR towards piperacillin (32% difference), tobramycin (24% difference), cefepime (19% difference), amikacin (12% difference), and meropenem (9% difference) in the Validation Dataset. In contrast, there was a greater proportion of false-positive and false-negative variants encoding amikacin AMR (5% difference) in the Global Dataset (Fig. 1).

Comparative genomic analysis of Validation Dataset isolates that yielded false-negative aminoglycoside AMR predictions identified that many belonged to a single multilocus sequence type (ST), ST801, also known as AUST-06. Among 23/24 aminoglycoside-AMR ST801 isolates, a clade-specific missense variant in elongation factor G (FusA1 S459F) was identified; this SNP was not observed in other Global or Validation Dataset isolates. The remaining aminoglycoside-AMR ST801 strain, SCHI0010.S.1, encoded *AAC*(6’)-IIa, an aminoglycoside-modifying enzyme. Inclusion of FusA1 S459F into our AMR database significantly increased ARDaP bACCs for the Validation Dataset by an average 19% for both amikacin and tobramycin, raising them to 95% and 90%, respectively, with no impact on Global Dataset bACC.

### Precision and recall among AMR software

**Fig. 2:**
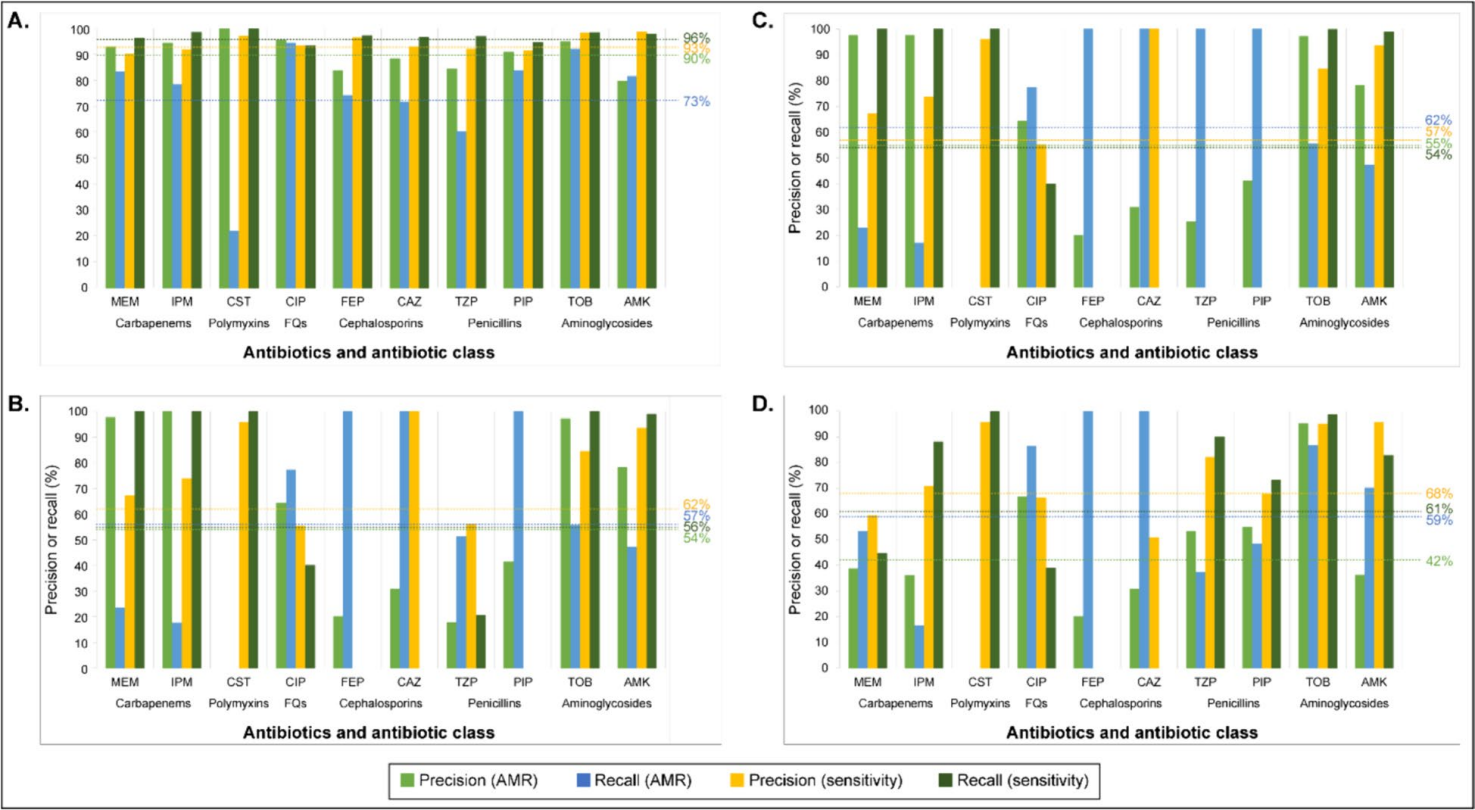
Precision and recall of ARDaP, abritAMR, AMRFinderPlus, and ResFinder software across the Global Dataset (*n* = 1877 strains). Precision and recall metrics for both antimicrobial-sensitive and antimicrobial-resistant (AMR) strains were highest using ARDaP (**A**; range 73–96%) vs. abritAMR (**B**; range 54–62%), AMRFinderPlus (**C**; range 54–62%) and ResFinder (**D**; range 42–68%). To enable software comparisons, isolates with intermediate resistance were removed prior to analysis. Abbreviations: AMK, amikacin; CAZ, ceftazidime; CIP, ciprofloxacin; CST, colistin; FEP, cefepime; FQs, fluoroquinolones; IPM, imipenem; MEM, meropenem; PIP, piperacillin; TZP, piperacillin/tazobactam; TOB, tobramycin. N.B. Precision (AMR) is also known as positive predictive value, and precision (sensitivity) is also known as negative predictive value

ARDaP demonstrated excellent precision and recall for predicting antimicrobial-sensitive and AMR phenotypes across the Global Dataset, ranging from 73% (average AMR recall) to 96% (average sensitivity recall) (Fig. 2A). In contrast, abritAMR ranged from 54% (average AMR precision) to 62% (average sensitivity precision) (Fig. 2B), AMRFinderPlus ranged from 54% (average sensitivity recall) to 62% (average AMR recall) (Fig. 2C), and ResFinder ranged from only 42% (average AMR precision) to 68% (average sensitivity precision) (Fig. 2D).

abritAMR, AMRFinderPlus, and ResFinder all yielded AMR precision and recall values of 0% for colistin; in other words, none of these tools identified colistin AMR variants in strains exhibiting a colistin AMR phenotype. In comparison, ARDaP identified colistin AMR strains with 21% recall and 100% precision. Similarly, abritAMR, AMRFinderPlus, and ResFinder all failed to predict cefepime sensitivity in any cefepime-sensitive strain (Fig. 2B–D), instead erroneously classing every strain as cefepime-resistant, whereas ARDaP correctly identified cefepime-sensitive strains with 96% precision and 96% recall (Fig. 2A). All three tools also failed to predict ceftazidime sensitivity. In addition, abritAMR and AMRFinderPlus failed to identify piperacillin sensitivity in any of the tested strains, and AMRFinderPlus failed to identify piperacillin/tazobactam sensitivity (Fig. 2B and Fig. 2C).

### Predictive performance for intermediate resistance

The inclusion of isolates with intermediate resistance reduced ARDaP bACCs by between 1 and 13% (6% average), depending on the antibiotic (Table S5). Intermediate resistance inclusion was most detrimental to cefepime AMR prediction (−13%), followed by imipenem (−8%), meropenem, ciprofloxacin, and ceftazidime (−7%), piperacillin/tazobactam and amikacin (−5%), piperacillin (−4%), and tobramycin (−1%). Colistin prediction was unchanged as no intermediate category exists for this antibiotic.

## Discussion

The increasing role of high-throughput sequencing in the clinic has driven the concomitant development of bioinformatic tools for AMR variant detection and antimicrobial phenotype prediction [113]. However, current gold standard AMR tools are limited in their accuracy and performance due to their heavy focus on AMR gene gain rather than AMR-conferring chromosomal variants and their inability to detect the gamut of genetic mutations that can confer AMR (i.e. indels, CNVs, large deletions, frameshift mutations, and structural variants) [14]. In addition, most tools have primarily focused on AMR gene detection rather than AMR phenotype prediction. These shortcomings become acutely evident when attempting to predict AMR in pathogens with complex resistomes like *P. aeruginosa* [8].

To address this issue, we first constructed a comprehensive and accurate database of AMR variants encoded by *P. aeruginosa*. Our database of 728 chromosomal variants (Dataset 1) comprises 362 previously identified variants that we confirmed were significantly associated with one or more AMR phenotypes, 75 previously unreported AMR-conferring variants (Table 2), 281 variants that we classed as natural variants due to their non-significant association with AMR strains (Table S3), and 10 loci that conferred unusual antimicrobial susceptibility. Natural variants were included in our database for three reasons: (i) to allow ARDaP’s coverage algorithm to scan known AMR genes for novel, high-consequence mutations (e.g. frameshift mutations in *oprD* that lead to carbapenem AMR) whilst avoiding variants that do not impact function, (ii) to substantially reduce the legwork involved in identifying putative novel AMR variants using mGWAS, machine learning, or comparative genomics, and (iii) to provide a valuable resource for minimising erroneous AMR variant reporting in future *P. aeruginosa* AMR variant discovery studies.

Next, performance assessment of our ARDaP-compatible *P. aeruginosa* AMR database against the Global and Validation Datasets showed that our tool outstripped the predictive performance of current ‘gold standard’ AMR software across all 10 antibiotics, yielding average bACCs of 85% and 81%, respectively. In comparison, average bACCs for the Global and Validation Datasets were just 56% and 54% for abriTAMR, 58% and 54% for AMRFinderPlus, and 60% and 53% for ResFinder, respectively (Fig. 1). This performance difference is due to our chromosomal AMR variant database, coupled with our comprehensive comparative genomics pipeline, which identified all types of chromosomal variation and linked these variants with individual antibiotic phenotypes (Dataset 1). ARDaP also demonstrated superior precision and recall metrics for both antimicrobial-sensitive and AMR strains across all 10 tested antibiotics (Fig. 2). Our findings concur with a recent study of 654 *P. aeruginosa* genomes, which also found that CARD and ResFinder exhibited poor AMR prediction performance metrics across all 11 tested anti-pseudomonal antibiotics [8].

Although still inferior to ARDaP, abritAMR, AMRFinderPlus, and ResFinder performed best when predicting aminoglycoside phenotypes in the Global Dataset (Fig. 1A), which is heavily populated with American and European strains (Table S1). However, these tools performed substantially worse when tested against the Australian Validation Dataset (Fig. 1B), with the average aminoglycoside bACC dropping by 16% for AMRFinderPlus and 26% for ResFinder. ARDaP’s bACC also initially dropped by 18% for the aminoglycosides. Upon closer inspection, we found that this performance reduction was predominantly due to false-negative calls among the ST801 isolates, a geographically restricted clone that has only been reported in people with CF in Qld, Australia [114]. Inclusion of one novel *fusA1* variant, identified with comparative genomics, restored ARDaP’s bACCs to 90% and 95% for amikacin and tobramycin, respectively. This performance difference across isolate datasets can be attributed to two phenomena. The first is the predominance of aminoglycoside-modifying enzymes in the Global (33%) but not Validation (8%) Datasets, reflecting potential major differences in the geographic prevalence of these enzymes that requires further exploration. The second is the enrichment of CF-derived isolates in the Validation Dataset, which comprise 86% of the aminoglycoside AMR strains (Fig S1). These isolates have largely developed aminoglycoside AMR via chromosomal mutation rather than aminoglycoside-modifying enzyme acquisition; as such, abritAMR, AMRFinderPlus, and ResFinder exhibited poor aminoglycoside AMR predictive capacity due to their limited chromosomal AMR variant databases. These performance differences highlight the need for including isolates from diverse sources, disease states, and locales to provide the most relevant AMR prediction software benchmarking comparisons. Our results suggest that abritAMR, AMRFinderPlus, and ResFinder are not useful for predicting aminoglycoside AMR from CF-derived *P. aeruginosa*, particularly in the Australian context, although this finding requires further exploration across larger, geographically diverse datasets.

Our findings revealed important weaknesses in abritAMR, AMRFinderPlus, and ResFinder when used for phenotype prediction. All three tools yielded bACCs of just 50% for cephalosporin prediction, abritAMR and AMRFinderPlus yielded a bACC of just 50% for piperacillin (Fig. 1A and Fig. 1B), and abritAMR performed worse than a coin flip for predicting piperacillin/tazobactam phenotypes, with a bACC of just 35%. This under-performance was largely attributed to sensitive isolates being predicted as AMR (Fig. 2). The inferior performance of abritAMR and AMRFinderPlus over ResFinder was further exacerbated by their software design; for most anti-pseudomonal antibiotics, these tools only predict phenotypes to the antibiotic class level. To facilitate direct software comparisons, AMR identified for a given antibiotic class by abritAMR and AMRFinderPlus was extrapolated to all antibiotics within that class, which likely led to higher imprecision or error due to differences in within-class antibiotic spectrum of activity. For instance, the poor abritAMR piperacillin/tazobactam bACC may be attributed to this tool only reporting ‘β-lactamase’ presence; however, the impact of this β-lactamase on piperacillin/tazobactam efficacy is not explicitly reported due to insufficient granularity. Based on our and other’s [8] collective findings, we strongly discourage the use of abritAMR, AMRFinderPlus, or ResFinder for in silico cephalosporin AMR prediction in *P. aeruginosa* as none of these tools are currently capable of accurately differentiating sensitive from AMR strains for these antibiotics. Furthermore, abritAMR and AMRFinderPlus should not be used to predict penicillin susceptibility phenotypes in *P. aeruginosa* due to their insufficient resolution.

Colistin prediction proved the most challenging of the 10 tested antibiotics, yielding bACCs of 50% with abriTAMR, AMRFinderPlus, and ResFinder, and 60% with ARDaP (Fig. 1). ARDaP was the only tool capable of correctly predicting some colistin AMR strains in the Global Dataset (Fig. 2A); the other three tools erroneously classified all *P. aeruginosa* strains as colistin-sensitive (Fig. 2B–2D). Accurate colistin prediction may have been hampered by the purported unreliability of gradient diffusion methods (e.g. disc diffusions and ETESTs) to accurately measure colistin breakpoints due to poor antibiotic diffusion and Mueller-Hinton agar manufacturer differences [115, 116]. In any case, our study highlights a major gap in understanding the basis of colistin AMR, and underscores the need for much more work in this area, especially given the increasing use of inhaled colistin in treatment-refractory, multidrug-resistant *P. aeruginosa* infections [117, 118].

Loss-of-function mutations affecting the specialised porin, OprD, are the most common cause of carbapenem AMR in *P. aeruginosa*, particularly in clinical isolates [9]. Although *oprD* is notoriously hypervariable [119], ARDaP’s ability to accurately identify functional OprD loss accounted for its high carbapenem bACCs (average of 88% and 84% in the Global and Validation Datasets, respectively, vs. just 60% and 55% for abritAMR, 59% and 55% for AMRFinderPlus, and 50% and 49% for ResFinder, respectively; Fig. 1). This outcome highlights the complex nature of the *P. aeruginosa* resistome, the necessity of manual AMR variant curation efforts, the value of AMR prediction tools that can accurately detect the spectrum of chromosomal variants, and the need for species-specific AMR variant databases to achieve the most accurate AMR predictions.

Mutations leading to chromosomal cephalosporinase (*ampC*) overexpression are an important cause of β-lactam AMR [15, 120]. To predict *ampC* upregulation, our database includes function-altering mutations in genes known to directly or indirectly regulate *ampC*. These chromosomally encoded variants, alongside acquired cephalosporinases, accounted for the higher average ARDaP bACC observed for cephalosporins (84% and 70% in the Global and Validation Datasets, respectively, vs. just 50% with abritAMR, AMRFinderPlus, and ResFinder across both Datasets; Fig. 1). The prominence of *ampC* overexpression-associated variants provides further support that this mechanism is a major cause of acquired cephalosporin AMR, particularly in clinical *P. aeruginosa* isolates [120, 121]. In support of this hypothesis, Khaledi and colleagues demonstrated a considerably higher bACC for ceftazidime AMR prediction when using both transcriptomic and genomic data (82%) compared with just genomic data (67%) [9]. Genomics alone cannot currently identify all instances of *ampC* over-expression, either because up-regulation is the result of an epigenic change or the mutation remains cryptic due to an incomplete understanding of *ampC* regulatory mechanisms. Indeed, a review of intrinsic β-lactamases by Juan and colleagues details the complexity of *ampC* expression and its intricate regulation, along with the challenge of corresponding elevated β-lactamase MICs driven by *ampC* upregulation to clinical AMR breakpoints [122]. Using a combination of genomic and transcriptomic data will likely lead to further improvements in AMR prediction for most antibiotics [9, 120].

Predicting intermediate resistance is exceedingly difficult from genomic data alone, even with complex machine learning algorithms that combine transcriptomic and genomic data [9]. We also encountered difficulties in predicting intermediate resistance, with the inclusion of intermediate strains dropping bACCs by up to 13% (Table S5). Possible explanations include the need to understand the contribution of stepwise variants in conferring decreased antibiotic susceptibility [15, 65, 123], subtle and rapidly reversible gene expression alterations [9] caused by methylation [124] or dynamic environmental stimuli, and undetected strain mixtures. Further refinement of our ARDaP-compatible database, such as capacity to analyse RNA-seq data, will continue to improve this critical yet understudied area. Nevertheless, the pioneering capacity of ARDaP to predict intermediate resistance, including stepwise mutations that lower the barrier to full AMR development, and to differentiate strain mixtures in metagenomic data has important implications for detecting emerging AMR in *P. aeruginosa* and informing earlier treatment shifts [14].

Errors introduced during sample collection, metadata curation, specimen processing, or sequencing may be partially responsible for our inability to predict AMR with a 100% bACC for any antibiotic. For example, 11 strains in the Khaledi et al. dataset [9] possessed variants known to confer ceftazidime AMR (e.g. *bla*_VIM-_[2, 4, 45], *bla*_OXA-2_, *bla*_GES-_[1, 5]) yet were reported as ceftazidime-sensitive, and 22 strains in the Kos et al. dataset were amikacin-sensitive, yet possessed the aminoglycoside-modifying enzyme gene *aac(6')-Ib-cr*, known to cause amikacin AMR and reduced ciprofloxacin susceptibility [125]. Due to the presence of these known AMR variants, all tools identified these strains as AMR, contributing to imperfect bACC (Fig. 1) and poor precision (Fig. 2) for amikacin and ceftazidime. As we did not have access to these strains, it was not possible to retest their AMR phenotypes or to repeat genome sequencing; however, we hypothesise that these strains would generate different results upon retesting. Alternatively, these AMR variants may be present but functionally or transcriptionally inactive, resulting in false-positive predictions for these isolates that must be factored into future AMR prediction estimates.

We recognise several study limitations. First, some false-positive predictions were identified across all antibiotic classes with our ARDaP-compatible database; however, these rates were significantly lower than those reported by other software (Fig. 1). Whilst not ideal, we chose to retain a small number of AMR variants that result in low-frequency false-positive predictions as (i) some strains may have reverted to a sensitive phenotype, despite encoding a known AMR variant, and (ii) we included phenotypic data generated by others, which may harbour inaccuracies. Functional profiling will be essential to fully understand the contribution of each of these variants in conferring AMR. Second, although we aimed for a phylogenetically diverse Validation Dataset (Fig. S1, Fig. S2), only isolates from Queensland, Australia, were included in this dataset, limiting geographic and genetic representation. Despite this shortcoming, the Validation Dataset proved extremely useful for identifying AMR variant database deficits across all three AMR tools, particularly those variants encoding AMR towards the aminoglycosides, piperacillin, cefepime, and meropenem, highlighting clear areas of need for future research efforts. Third, due to cost constraints, we only performed ciprofloxacin and meropenem ETESTs for the Validation Dataset isolates, with disc diffusions used for the remaining eight antibiotics, a less robust methodology that may have led to some minor discrepancies in antimicrobial phenotype assignments. Fourth, we did not test ARDaP’s capacity to identify *P. aeruginosa* AMR variants from simulated or real metagenomic datasets and strain mixtures as we have demonstrated this capacity elsewhere [14, 103], nor did we compare software performance against ARESdb [126] due to the proprietary nature of this database. Fifth, we recognise the importance of functional studies to fully validate new AMR variants such as those listed in Table 2, although these experiments are laborious, time-consuming, and rarely straightforward, and as such, were not conducted as part of this study. Somewhat unsurprisingly, most of these newly discovered mutations occur in genes with already well-characterised roles in driving AMR, and have been the subject of previous functional studies (e.g. *oprD* [22]), making their role in conferring AMR less contentious. Finally, our study would have benefitted from the inclusion of transcriptomic data [9] to identify additional novel variants associated with AMR. Although our understanding of the molecular mechanisms of AMR in *P. aeruginosa* is improving rapidly in the genomics era, false-negative predictions still occur across all antibiotic classes. Using ARDaP, such false-negative strains can now be rapidly identified and targeted for future functional work to pinpoint novel AMR variants and mechanisms.

## Conclusions

Improved AMR diagnostics, more personalised treatment regimens, and better-informed antimicrobial stewardship measures are crucial for tackling the impending AMR crisis. To this end, we developed a comprehensive *P. aeruginosa* AMR database that, when used in conjunction with our freely available ARDaP software, predicts AMR towards first- and second-line anti-pseudomonal antibiotics from (meta)genomic data with > 80% accuracy. In comparison, other freely available AMR software can only predict AMR in this pathogen with ≤ 60% accuracy. Our tool generates a clinician-friendly report that predicts antimicrobial susceptibility across 10 anti-pseudomonal antibiotics, enabling it to be readily incorporated into genomics workflows to enhance the diagnosis and surveillance of emerging and circulating *P. aeruginosa* AMR strains. To improve AMR prediction performance, more functional work is needed to capture the full breadth of genetic and transcriptional changes driving AMR development in this superbug.

### Supplementary Information

Additional file 1:* Pseudomonas aeruginosa *ARDaP-compatible antimicrobial resistance variant database.

Additional file 2: Fig. S1. Maximum likelihood phylogeny of ‘Validation Dataset’ isolates, their AMR profiles, and their disease origin. Fig. S2. Maximum likelihood phylogeny of all strains examined in this study. Fig. S3. Example clinician-friendly report produced by ARDaP. Table S1: Global Dataset strains, and their paired AMR and genomic data; Table S2: Validation Dataset strains generated in this study, and their AMR profiles. Table S3: Natural variants not associated with conferring AMR. Table S4: AMR rates in the Validation Dataset. Table S5: ARDaP performance across the Global Dataset when including intermediate resistance phenotype isolates.

## Data Availability

The datasets supporting the conclusions of this article are available at the NCBI Genome website under the accession numbers GCF_000006765.1 (https://www.ncbi.nlm.nih.gov/datasets/genome/GCF_000006765.1/) [19], GCF_003839205.1 (https://www.ncbi.nlm.nih.gov/datasets/genome/GCF_003839205.1/) [102], and GCF_003839985.1 (https://www.ncbi.nlm.nih.gov/datasets/genome/GCF_003839985.1/) [102] and the NCBI Sequence Read Archive database via BioProject accessions PRJEB15036 (https://www.ncbi.nlm.nih.gov/bioproject/?term=PRJEB15036) [50], PRJEB21341 (https://www.ncbi.nlm.nih.gov/bioproject/?term=PRJEB21341) [52], PRJEB29539 (https://www.ncbi.nlm.nih.gov/bioproject/?term=PRJEB29539) [94], PRJEB14771 (https://www.ncbi.nlm.nih.gov/bioproject/?term=PRJEB14771) [31], PRJNA297679 (https://www.ncbi.nlm.nih.gov/bioproject/?term=PRJNA297679) [93], PRJNA317143 (https://www.ncbi.nlm.nih.gov/bioproject/?term=PRJNA317143) [95], PRJNA526797 (https://www.ncbi.nlm.nih.gov/bioproject/?term=PRJNA526797) [9], PRJNA264310 (https://www.ncbi.nlm.nih.gov/bioproject/?term=PRJNA264310) [21], PRJNA388357 (https://www.ncbi.nlm.nih.gov/bioproject/?term=PRJNA388357) [96], PRJNA793523 (https://www.ncbi.nlm.nih.gov/bioproject/?term=PRJNA793523) [99], PRJEB40140 (https://www.ncbi.nlm.nih.gov/bioproject/?term=PRJEB40140) [98], PRJNA542028 (https://www.ncbi.nlm.nih.gov/bioproject/?term=PRJNA542028) [28], and PRJNA532924 (https://www.ncbi.nlm.nih.gov/bioproject/?term=PRJNA532924) [97] (Global Dataset). The *P. aeruginosa* AMR database constructed for this study (https://github.com/dsarov/ARDaP/tree/master/Databases/Pseudomonas_aeruginosa_pao1) [18] and the ARDaP software (https://github.com/dsarov/ARDaP) [18] are both freely available. Additional code used in this work is available at (https://github.com/dsarov/P_aeruginosa_ARDaP_manuscript) [127].

## References

[1] Mancuso G, Midiri A, Gerace E, Biondo C (2021). Bacterial antibiotic resistance: the most critical pathogens. Pathogens (Basel, Switzerland)..

[2] Bassetti M, Merelli M, Temperoni C, Astilean A (2013). New antibiotics for bad bugs: where are we?. Annals of Clinical Microbiology and Antimicrobials..

[3] Alanis AJ (2005). Resistance to antibiotics: are we in the post-antibiotic era?. Archives of Medical Research..

[4] O'Neill JC. Antimicrobial resistance: tackling a crisis for the health and wealth of nations 2014. https://amr-review.org/sites/default/files/AMR%20Review%20Paper%20-%20Tackling%20a%20crisis%20for%20the%20health%20and%20wealth%20of%20nations_1.pdf. Accessed 1 Aug 2022.

[5] G. B. D (2022). Antimicrobial Resistance Collaborators. Global mortality associated with 33 bacterial pathogens in 2019 a systematic analysis for the Global Burden of Disease Study 2019. Lancet.

[6] Denissen J, Reyneke B, Waso-Reyneke M, Havenga B, Barnard T, Khan S, Khan W (2022). Prevalence of ESKAPE pathogens in the environment: antibiotic resistance status, community-acquired infection and risk to human health. Int J Hyg Environ Health..

[7] Haenni M, Hocquet D, Ponsin C, Cholley P, Guyeux C, Madec JY, Bertrand X (2015). Population structure and antimicrobial susceptibility of *Pseudomonas aeruginosa* from animal infections in France. BMC Vet Res..

[8] Mahfouz N, Ferreira I, Beisken S, von Haeseler A, Posch AE (2020). Large-scale assessment of antimicrobial resistance marker databases for genetic phenotype prediction: a systematic review. Journal of Antimicrobial Chemotherapy..

[9] Khaledi A, Weimann A, Schniederjans M, Asgari E, Kuo TH, Oliver A (2020). Predicting antimicrobial resistance in *Pseudomonas aeruginosa* with machine learning-enabled molecular diagnostics. EMBO Mol Med..

[10] Bortolaia V, Kaas RS, Ruppe E, Roberts MC, Schwarz S, Cattoir V (2020). ResFinder 4.0 for predictions of phenotypes from genotypes. Journal of Antimicrobial Chemotherapy..

[11] Alcock BP, Raphenya AR, Lau TTY, Tsang KK, Bouchard M, Edalatmand A (2019). CARD 2020: antibiotic resistome surveillance with the comprehensive antibiotic resistance database. Nucleic Acids Research..

[12] Hunt M, Mather AE, Sanchez-Buso L, Page AJ, Parkhill J, Keane JA, Harris SR (2017). ARIBA: Rapid antimicrobial resistance genotyping directly from sequencing reads. Microbial genomics..

[13] Zankari E, Allesoe R, Joensen KG, Cavaco LM, Lund O, Aarestrup FM (2017). PointFinder: a novel web tool for WGS-based detection of antimicrobial resistance associated with chromosomal point mutations in bacterial pathogens. Journal of Antimicrobial Chemotherapy..

[14] Madden DE, Webb JR, Steinig EJ, Currie BJ, Price EP, Sarovich DS (2021). Taking the next-gen step: comprehensive antimicrobial resistance detection from *Burkholderia pseudomallei*. EBioMedicine..

[15] Lister PD, Wolter DJ, Hanson ND (2009). Antibacterial-resistant Pseudomonas aeruginosa: clinical impact and complex regulation of chromosomally encoded resistance mechanisms. Clin Microbiol Rev..

[16] Pesesky MW, Hussain T, Wallace M, Patel S, Andleeb S, Burnham CD, Dantas G (2016). Evaluation of machine learning and rules-based approaches for predicting antimicrobial resistance profiles in Gram-negative Bacilli from whole genome sequence data. Frontiers in microbiology..

[17] Gupta SK, Padmanabhan BR, Diene SM, Lopez-Rojas R, Kempf M, Landraud L, Rolain JM (2014). ARG-ANNOT, a new bioinformatic tool to discover antibiotic resistance genes in bacterial genomes. Antimicrobial agents and chemotherapy..

[18] Sarovich DS, Madden DE, Price EP (2024). ARDaP - Antimicrobial Resistance Detection and Prediction..

[19] Stover CK, Pham XQ, Erwin AL, Mizoguchi SD, Warrener P, Hickey MJ (2000). Complete genome sequence of *Pseudomonas aeruginosa* PAO1, an opportunistic pathogen. Nature..

[20] Lopez-Causape C, Cabot G, Del Barrio-Tofino E, Oliver A (2018). The versatile mutational resistome of *Pseudomonas aeruginosa*. Frontiers in microbiology..

[21] Kos VN, Deraspe M, McLaughlin RE, Whiteaker JD, Roy PH, Alm RA (2015). The resistome of *Pseudomonas aeruginosa* in relationship to phenotypic susceptibility. Antimicrobial agents and chemotherapy..

[22] Poole K (2011). *Pseudomonas aeruginosa*: resistance to the max. Frontiers in microbiology..

[23] Richardot C, Plesiat P, Fournier D, Monlezun L, Broutin I, Llanes C (2015). Carbapenem resistance in cystic fibrosis strains of *Pseudomonas aeruginosa* as a result of amino acid substitutions in porin OprD. International journal of antimicrobial agents..

[24] Tomás M, Doumith M, Warner M, Turton JF, Beceiro A, Bou G (2010). Efflux pumps, OprD porin, AmpC beta-lactamase, and multiresistance in *Pseudomonas aeruginosa* isolates from cystic fibrosis patients. Antimicrobial agents and chemotherapy..

[25] Heffernan AJ, Sime FB, Sarovich DS, Neely M, Guerra-Valero Y, Naicker S (2020). Pharmacodynamic evaluation of plasma and epithelial lining fluid exposures of amikacin against *Pseudomonas aeruginosa* in a dynamic *in vitro* hollow-fiber infection model. Antimicrobial agents and chemotherapy..

[26] Lau CH, Krahn T, Gilmour C, Mullen E, Poole K (2015). AmgRS-mediated envelope stress-inducible expression of the *mexXY* multidrug efflux operon of *Pseudomonas aeruginosa*. MicrobiologyOpen..

[27] Bolard A, Plésiat P, Jeannot K (2018). Mutations in gene *fusA1* as a novel mechanism of aminoglycoside resistance in clinical strains of *Pseudomonas aeruginosa*. Antimicrobial agents and chemotherapy..

[28] Wardell SJT, Rehman A, Martin LW, Winstanley C, Patrick WM, Lamont IL (2019). A large-scale whole-genome comparison shows that experimental evolution in response to antibiotics predicts changes in naturally evolved clinical *Pseudomonas aeruginosa*. Antimicrob Agents Chemother.

[29] López-Causapé C, Sommer LM, Cabot G, Rubio R, Ocampo-Sosa AA, Johansen HK (2017). Evolution of the *Pseudomonas aeruginosa* mutational resistome in an international cystic fibrosis clone. Sci Rep..

[30] Lopez-Causape C, Rubio R, Cabot G, Oliver A (2018). Evolution of the *Pseudomonas aeruginosa* aminoglycoside mutational resistome *in vitro* and in the cystic fibrosis setting. Antimicrobial agents and chemotherapy..

[31] Sherrard LJ, Tai AS, Wee BA, Ramsay KA, Kidd TJ, Ben Zakour NL (2017). Within-host whole genome analysis of an antibiotic resistant *Pseudomonas aeruginosa* strain sub-type in cystic fibrosis. PLoS One..

[32] Feng Y, Jonker MJ, Moustakas I, Brul S, Ter Kuile BH (2016). Dynamics of mutations during development of resistance by *Pseudomonas aeruginosa* against five antibiotics. Antimicrobial agents and chemotherapy..

[33] Sanz-Garcia F, Hernando-Amado S, Martinez JL (2018). Mutational evolution of *Pseudomonas aeruginosa* resistance to ribosome-targeting antibiotics. Front Genet..

[34] Schurek KN, Marr AK, Taylor PK, Wiegand I, Semenec L, Khaira BK, Hancock RE (2008). Novel genetic determinants of low-level aminoglycoside resistance in *Pseudomonas aeruginosa*. Antimicrobial agents and chemotherapy..

[35] Sun E, Gill EE, Falsafi R, Yeung A, Liu S, Hancock REW (2018). Broad-spectrum adaptive antibiotic resistance associated with *Pseudomonas aeruginosa* mucin-dependent surfing motility. Antimicrobial agents and chemotherapy..

[36] Dotsch A, Becker T, Pommerenke C, Magnowska Z, Jansch L, Haussler S (2009). Genomewide identification of genetic determinants of antimicrobial drug resistance in *Pseudomonas aeruginosa*. Antimicrob Agents Chemother.

[37] Berrazeg M, Jeannot K, Ntsogo Enguene VY, Broutin I, Loeffert S, Fournier D, Plesiat P (2015). Mutations in beta-Lactamase *ampC* increase resistance of *Pseudomonas aeruginosa* isolates to antipseudomonal cephalosporins. Antimicrobial agents and chemotherapy..

[38] Colque CA, Albarracín Orio AG, Feliziani S, Marvig RL, Tobares AR, Johansen HK (2020). Hypermutator *Pseudomonas aeruginosa* exploits multiple genetic pathways to develop multidrug resistance during long-term infections in the airways of cystic fibrosis patients. Antimicrobial agents and chemotherapy..

[39] Slater CL, Winogrodzki J, Fraile-Ribot PA, Oliver A, Khajehpour M, Mark BL (2020). Adding insult to injury: mechanistic basis for how AmpC mutations allow *Pseudomonas aeruginosa* to accelerate cephalosporin hydrolysis and evade avibactam. Antimicrobial agents and chemotherapy..

[40] Fernandez-Esgueva M, Lopez-Calleja AI, Mulet X, Fraile-Ribot PA, Cabot G, Huarte R (2020). Characterization of AmpC beta-lactamase mutations of extensively drug-resistant *Pseudomonas aeruginosa* isolates that develop resistance to ceftolozane/tazobactam during therapy. Enferm Infecc Microbiol Clin (Engl Ed)..

[41] Moya B, Dotsch A, Juan C, Blazquez J, Zamorano L, Haussler S, Oliver A (2009). Beta-lactam resistance response triggered by inactivation of a nonessential penicillin-binding protein. PLoS Pathog..

[42] Zamorano L, Reeve TM, Deng L, Juan C, Moya B, Cabot G (2010). NagZ inactivation prevents and reverts beta-lactam resistance, driven by AmpD and PBP 4 mutations. Pseudomonas aeruginosa. Antimicrobial agents and chemotherapy..

[43] Barbosa C, Gregg KS, Woods RJ (2020). Variants in *ampD* and *dacB* lead to *in vivo* resistance evolution of *Pseudomonas aeruginosa* within the central nervous system. Journal of Antimicrobial Chemotherapy..

[44] Langaee TY, Gagnon L, Huletsky A (2000). Inactivation of the *ampD* gene in *Pseudomonas aeruginosa* leads to moderate-basal-level and hyperinducible AmpC beta-lactamase expression. Antimicrobial agents and chemotherapy..

[45] Juan C, Macia MD, Gutierrez O, Vidal C, Perez JL, Oliver A (2005). Molecular mechanisms of beta-lactam resistance mediated by AmpC hyperproduction in *Pseudomonas aeruginosa* clinical strains. Antimicrobial agents and chemotherapy..

[46] Bagge N, Ciofu O, Hentzer M, Campbell JI, Givskov M, Hoiby N (2002). Constitutive high expression of chromosomal beta-lactamase in *Pseudomonas aeruginosa* caused by a new insertion sequence (IS1669) located in *ampD*. Antimicrobial agents and chemotherapy..

[47] Quale J, Bratu S, Gupta J, Landman D (2006). Interplay of efflux system, *ampC*, and *oprD* expression in carbapenem resistance of *Pseudomonas aeruginosa* clinical isolates. Antimicrobial agents and chemotherapy..

[48] Tsutsumi Y, Tomita H, Tanimoto K (2013). Identification of novel genes responsible for overexpression of *ampC* in *Pseudomonas aeruginosa* PAO1. Antimicrobial agents and chemotherapy..

[49] Kong KF, Aguila A, Schneper L, Mathee K (2010). *Pseudomonas aeruginosa* β-lactamase induction requires two permeases. AmpG and AmpP. BMC microbiology..

[50] Cabot G, Lopez-Causape C, Ocampo-Sosa AA, Sommer LM, Dominguez MA, Zamorano L (2016). Deciphering the resistome of the widespread *Pseudomonas aeruginosa* sequence type 175 international high-risk clone through whole-genome sequencing. Antimicrobial agents and chemotherapy..

[51] Clark ST, Sinha U, Zhang Y, Wang PW, Donaldson SL, Coburn B (2019). Penicillin-binding protein 3 is a common adaptive target among *Pseudomonas aeruginosa* isolates from adult cystic fibrosis patients treated with beta-lactams. Int J Antimicrob Agents.

[52] Del Barrio-Tofiño E, López-Causapé C, Cabot G, Rivera A, Benito N, Segura C (2017). Genomics and susceptibility profiles of extensively drug-resistant *Pseudomonas aeruginosa* isolates from Spain. Antimicrobial agents and chemotherapy..

[53] Sherrard LJ, Wee BA, Duplancic C, Ramsay KA, Dave KA, Ballard E (2021). Emergence and impact of *oprD* mutations in *Pseudomonas aeruginosa* strains in cystic fibrosis. J Cyst Fibros Soc..

[54] Livermore DM (1992). Interplay of impermeability and chromosomal beta-lactamase activity in imipenem-resistant *Pseudomonas aeruginosa*. Antimicrobial agents and chemotherapy..

[55] Ocampo-Sosa AA, Cabot G, Rodriguez C, Roman E, Tubau F, Macia MD (2012). Alterations of OprD in carbapenem-intermediate and -susceptible strains of *Pseudomonas aeruginosa* isolated from patients with bacteremia in a Spanish multicenter study. Antimicrobial agents and chemotherapy..

[56] Fraile-Ribot PA, Mulet X, Cabot G, Del Barrio-Tofino E, Juan C, Perez JL, Oliver A (2017). *In vivo* emergence of resistance to novel cephalosporin-beta-lactamase inhibitor combinations through the duplication of amino acid D149 from OXA-2 beta-lactamase (OXA-539) in sequence type 235 *Pseudomonas aeruginosa*. Antimicrob Agents Chemother..

[57] Cabot G, Bruchmann S, Mulet X, Zamorano L, Moyà B, Juan C (2014). *Pseudomonas aeruginosa* ceftolozane-tazobactam resistance development requires multiple mutations leading to overexpression and structural modification of AmpC. Antimicrobial agents and chemotherapy..

[58] Perron K, Caille O, Rossier C, Van Delden C, Dumas JL, Kohler T (2004). CzcR-CzcS, a two-component system involved in heavy metal and carbapenem resistance in *Pseudomonas aeruginosa*. J Biol Chem..

[59] Higgins PG, Fluit AC, Milatovic D, Verhoef J, Schmitz FJ (2003). Mutations in GyrA, ParC, MexR and NfxB in clinical isolates of *Pseudomonas aeruginosa*. International journal of antimicrobial agents..

[60] Lee JK, Lee YS, Park YK, Kim BS (2005). Alterations in the GyrA and GyrB subunits of topoisomerase II and the ParC and ParE subunits of topoisomerase IV in ciprofloxacin-resistant clinical isolates of *Pseudomonas aeruginosa*. International journal of antimicrobial agents..

[61] Bruchmann S, Dötsch A, Nouri B, Chaberny IF, Häussler S (2013). Quantitative contributions of target alteration and decreased drug accumulation to *Pseudomonas aeruginosa* fluoroquinolone resistance. Antimicrobial agents and chemotherapy..

[62] Jalal S, Wretlind B (1998). Mechanisms of quinolone resistance in clinical strains of *Pseudomonas aeruginosa*. Microbial drug resistance (Larchmont, NY)..

[63] Alyaseen SA, Piper KE, Rouse MS, Steckelberg JM, Patel R (2005). Selection of cross-resistance following exposure of *Pseudomonas aeruginosa* clinical isolates to ciprofloxacin or cefepime. Antimicrobial agents and chemotherapy..

[64] Akasaka T, Tanaka M, Yamaguchi A, Sato K (2001). Type II topoisomerase mutations in fluoroquinolone-resistant clinical strains of *Pseudomonas aeruginosa* isolated in 1998 and 1999: role of target enzyme in mechanism of fluoroquinolone resistance. Antimicrobial agents and chemotherapy..

[65] Rehman A, Jeukens J, Levesque RC, Lamont IL (2021). Gene-gene interactions dictate ciprofloxacin resistance in *Pseudomonas aeruginosa* and facilitate prediction of resistance phenotype from genome sequence data. Antimicrobial agents and chemotherapy..

[66] Chilam J, Argimon S, Limas MT, Masim ML, Gayeta JM, Lagrada ML (2021). Genomic surveillance of *Pseudomonas aeruginosa* in the Philippines, 2013–2014. Western Pac Surveill Response J..

[67] Lee JY, Ko KS (2014). Mutations and expression of PmrAB and PhoPQ related with colistin resistance in *Pseudomonas aeruginosa* clinical isolates. Diagn Microbiol Infect Dis..

[68] Moskowitz SM, Ernst RK, Miller SI (2004). PmrAB, a two-component regulatory system of *Pseudomonas aeruginosa* that modulates resistance to cationic antimicrobial peptides and addition of aminoarabinose to lipid A. J Bacteriol..

[69] Abraham N, Kwon DH (2009). A single amino acid substitution in PmrB is associated with polymyxin B resistance in clinical isolate of *Pseudomonas aeruginosa*. FEMS Microbiol Lett..

[70] Moskowitz SM, Brannon MK, Dasgupta N, Pier M, Sgambati N, Miller AK (2012). PmrB mutations promote polymyxin resistance of *Pseudomonas aeruginosa* isolated from colistin-treated cystic fibrosis patients. Antimicrobial agents and chemotherapy..

[71] Owusu-Anim D, Kwon DH. Differential role of two-component regulatory systems (*phoPQ* and *pmrAB*) in polymyxin B susceptibility of *Pseudomonas aeruginosa*. Adv Microbiol. 2012;2(1). 10.4236/aim.2012.21005. 10.4236/aim.2012.21005 PMC3859615 24349887

[72] Choi MJ, Ko KS (2014). Mutant prevention concentrations of colistin for *Acinetobacter baumannii, Pseudomonas aeruginosa* and *Klebsiella pneumoniae* clinical isolates. Journal of Antimicrobial Chemotherapy..

[73] Saito K, Akama H, Yoshihara E, Nakae T (2003). Mutations affecting DNA-binding activity of the MexR repressor of *mexR-mexA-mexB-oprM* operon expression. J Bacteriol..

[74] Vaez H, Safaei HG, Faghri J (2017). The emergence of multidrug-resistant clone ST664 *Pseudomonas aeruginosa* in a referral burn hospital, Isfahan. Iran. Burns Trauma..

[75] Braz VS, Furlan JP, Fernandes AF, Stehling EG (2016). Mutations in NalC induce MexAB-OprM overexpression resulting in high level of aztreonam resistance in environmental isolates of *Pseudomonas aeruginosa*. FEMS Microbiol Lett..

[76] Suresh M, Nithya N, Jayasree PR, Vimal KP, Manish Kumar PR (2018). Mutational analyses of regulatory genes, *mexR*, *nalC*, *nalD* and *mexZ* of *mexAB-oprM* and *mexXY* operons, in efflux pump hyperexpressing multidrug-resistant clinical isolates of *Pseudomonas aeruginosa*. World journal of microbiology & biotechnology..

[77] Yan J, Estanbouli H, Liao C, Kim W, Monk JM, Rahman R (2019). Systems-level analysis of NalD mutation, a recurrent driver of rapid drug resistance in acute *Pseudomonas aeruginosa* infection. PLoS Comput Biol..

[78] Sobel ML, Neshat S, Poole K (2005). Mutations in *PA2491* (*mexS*) promote MexT-dependent *mexEF-oprN* expression and multidrug resistance in a clinical strain of *Pseudomonas aeruginosa*. J Bacteriol..

[79] Juarez P, Broutin I, Bordi C, Plésiat P, Llanes C (2018). Constitutive activation of *mexT* by amino acid substitutions results in MexEF-OprN overproduction in clinical isolates of *Pseudomonas aeruginosa*. Antimicrob Agents Chemother..

[80] Llanes C, Köhler T, Patry I, Dehecq B, van Delden C, Plésiat P (2011). Role of the MexEF-OprN efflux system in low-level resistance of *Pseudomonas aeruginosa* to ciprofloxacin. Antimicrobial agents and chemotherapy..

[81] Guénard S, Muller C, Monlezun L, Benas P, Broutin I, Jeannot K, Plésiat P (2014). Multiple mutations lead to MexXY-OprM-dependent aminoglycoside resistance in clinical strains of *Pseudomonas aeruginosa*. Antimicrobial agents and chemotherapy..

[82] Chuanchuen R, Wannaprasat W, Ajariyakhajorn K, Schweizer HP (2008). Role of the MexXY multidrug efflux pump in moderate aminoglycoside resistance in *Pseudomonas aeruginosa* isolates from *Pseudomonas* mastitis. Microbiology and immunology..

[83] Hocquet D, Muller A, Blanc K, Plesiat P, Talon D, Monnet DL, Bertrand X (2008). Relationship between antibiotic use and incidence of MexXY-OprM overproducers among clinical isolates of *Pseudomonas aeruginosa*. Antimicrobial agents and chemotherapy..

[84] Islam S, Jalal S, Wretlind B (2004). Expression of the MexXY efflux pump in amikacin-resistant isolates of *Pseudomonas aeruginosa*. Clinical microbiology and infection : the official publication of the European Society of Clinical Microbiology and Infectious Diseases..

[85] Alvarez-Ortega C, Wiegand I, Olivares J, Hancock RE, Martinez JL (2011). The intrinsic resistome of *Pseudomonas aeruginosa* to beta-lactams. Virulence..

[86] Li XZ, Ma D, Livermore DM, Nikaido H (1994). Role of efflux pump(s) in intrinsic resistance of *Pseudomonas aeruginosa*: active efflux as a contributing factor to beta-lactam resistance. Antimicrobial agents and chemotherapy..

[87] Poole K, Heinrichs DE, Neshat S (1993). Cloning and sequence analysis of an EnvCD homologue in *Pseudomonas aeruginosa*: regulation by iron and possible involvement in the secretion of the siderophore pyoverdine. Mol Microbiol..

[88] Mine T, Morita Y, Kataoka A, Mizushima T, Tsuchiya T (1999). Expression in *Escherichia coli* of a new multidrug efflux pump, MexXY, from *Pseudomonas aeruginosa*. Antimicrobial agents and chemotherapy..

[89] Poole K, Gotoh N, Tsujimoto H, Zhao Q, Wada A, Yamasaki T (1996). Overexpression of the *mexC-mexD-oprJ* efflux operon in *nfxB*-type multidrug-resistant strains of *Pseudomonas aeruginosa*. Mol Microbiol..

[90] Köhler T, Michéa-Hamzehpour M, Henze U, Gotoh N, Curty LK, Pechère JC (1997). Characterization of MexE-MexF-OprN, a positively regulated multidrug efflux system of *Pseudomonas aeruginosa*. Mol Microbiol..

[91] Anuj SN, Whiley DM, Kidd TJ, Bell SC, Wainwright CE, Nissen MD, Sloots TP (2009). Identification of *Pseudomonas aeruginosa* by a duplex real-time polymerase chain reaction assay targeting the *ecfX* and the *gyrB* genes. Diagn Microbiol Infect Dis..

[92] Oliver A, Baquero F, Blázquez J (2002). The mismatch repair system (*mutS*, *mutL* and *uvrD* genes) in *Pseudomonas aeruginosa*: molecular characterization of naturally occurring mutants. Mol Microbiol..

[93] van Belkum A, Soriaga LB, LaFave MC, Akella S, Veyrieras JB, Barbu EM, et al. Phylogenetic distribution of CRISPR-Cas systems in antibiotic-resistant *Pseudomonas aeruginosa*. mBio. 2015;6(6):e01796-15. 10.1128/mBio.01796-15 PMC4669384 26604259

[94] Buhl M, Kästle C, Geyer A, Autenrieth IB, Peter S, Willmann M (2019). Molecular evolution of extensively drug-resistant (XDR) *Pseudomonas aeruginosa* strains from patients and hospital environment in a prolonged outbreak. Frontiers in microbiology..

[95] United States Centers for Disease Control and Prevention. CDC & FDA Antibiotic Resistance Isolate Bank for *Pseudomonas aeruginosa* Accessed 18Jan22 [Available from: Available at: https://wwwn.cdc.gov/ARIsolateBank/Panel/PanelDetail?ID=12.

[96] Ramanathan B, Jindal HM, Le CF, Gudimella R, Anwar A, Razali R (2017). Next generation sequencing reveals the antibiotic resistant variants in the genome of *Pseudomonas aeruginosa*. PLoS One..

[97] Tsang KK, Maguire F, Zubyk HL, Chou S, Edalatmand A, Wright GD (2021). Identifying novel β-lactamase substrate activity through *in silico* prediction of antimicrobial resistance. Microb Genom..

[98] Cortes-Lara S, Barrio-Tofiño ED, López-Causapé C, Oliver A, Gemara-Seimc Reipi Pseudomonas study Group. Predicting *Pseudomonas aeruginosa* susceptibility phenotypes from whole genome sequence resistome analysis. Clinical microbiology and infection : the official publication of the European Society of Clinical Microbiology and Infectious Diseases. 2021;27(11):1631-7. 10.1016/j.cmi.2021.05.011 34015532

[99] Sun Z, Yang F, Ji J, Cao W, Liu C, Ding B, Xu X (2023). Dissecting the genotypic features of a fluoroquinolone-resistant Pseudomonas aeruginosa ST316 sublineage causing ear infections in Shanghai, China. Microbial genomics..

[100] Su M, Satola SW, Read TD (2019). Genome-based prediction of bacterial antibiotic resistance. J Clin Microbiol.

[101] Madden DE, McCarthy KL, Bell SC, Olagoke O, Baird T, Neill J (2022). Rapid fluoroquinolone resistance detection in *Pseudomonas aeruginosa* using mismatch amplification mutation assay-based real-time PCR. J Med Microbiol..

[102] Freschi L, Vincent AT, Jeukens J, Emond-Rheault JG, Kukavica-Ibrulj I, Dupont MJ (2019). The *Pseudomonas aeruginosa* pan-genome provides new insights on its population structure, horizontal gene transfer, and pathogenicity. Genome Biol Evol..

[103] Madden DE, Olagoke O, Baird T, Neill J, Ramsay KA, Fraser TA (2022). Express yourself: Quantitative real-time PCR assays for rapid chromosomal antimicrobial resistance detection in *Pseudomonas aeruginosa*. Antimicrob Agents Chemother.

[104] Mobegi FM, Cremers AJ, de Jonge MI, Bentley SD, van Hijum SA, Zomer A (2017). Deciphering the distance to antibiotic resistance for the pneumococcus using genome sequencing data. Sci Rep..

[105] Sarovich DS, Price EP (2014). SPANDx: a genomics pipeline for comparative analysis of large haploid whole genome re-sequencing datasets. BMC research notes..

[106] Crouch DJM, Bodmer WF (2020). Polygenic inheritance, GWAS, polygenic risk scores, and the search for functional variants. Proc Natl Acad Sci U S A..

[107] Sherry NL, Horan KA, Ballard SA, Gonҫalves da Silva A, Gorrie CL, Schultz MB, et al. An ISO-certified genomics workflow for identification and surveillance of antimicrobial resistance. Nat Commun. 2023;14(1):60. 10.1038/s41467-022-35713-4 PMC9813266 36599823

[108] Feldgarden M, Brover V, Haft DH, Prasad AB, Slotta DJ, Tolstoy I (2019). Validating the AMRFinder tool and resistance gene database by using antimicrobial resistance genotype-phenotype correlations in a collection of isolates. Antimicrob Agents Chemother.

[109] Prasad A. Interpreting results 2023 [Available from: https://github.com/ncbi/amr/wiki/Interpreting-results#genotype-vs-phenotype.

[110] Rodríguez-Martínez JM, Poirel L, Nordmann P (2009). Extended-spectrum cephalosporinases in *Pseudomonas aeruginosa*. Antimicrob Agents Chemother.

[111] Bekkar M, Djemaa HK, Alitouche TA (2013). Evaluation measures for models assessment over imbalanced data sets. J Inf Eng Appl..

[112] Hicks AL, Wheeler N, Sanchez-Buso L, Rakeman JL, Harris SR, Grad YH (2019). Evaluation of parameters affecting performance and reliability of machine learning-based antibiotic susceptibility testing from whole genome sequencing data. PLoS Comput Biol..

[113] Van Goethem N, Descamps T, Devleesschauwer B, Roosens NHC, Boon NAM, Van Oyen H, Robert A (2019). Status and potential of bacterial genomics for public health practice: a scoping review. Implement Sci..

[114] Kidd TJ, Ritchie SR, Ramsay KA, Grimwood K, Bell SC, Rainey PB (2012). *Pseudomonas aeruginosa* exhibits frequent recombination, but only a limited association between genotype and ecological setting. PLoS One..

[115] Matuschek E, Ahman J, Webster C, Kahlmeter G (2018). Antimicrobial susceptibility testing of colistin - evaluation of seven commercial MIC products against standard broth microdilution for *Escherichia coli*, *Klebsiella pneumoniae*, *Pseudomonas aeruginosa*, and *Acinetobacter* spp. Clinical microbiology and infection : the official publication of the European Society of Clinical Microbiology and Infectious Diseases..

[116] Bassetti M, Vena A, Croxatto A, Righi E, Guery B (2018). How to manage *Pseudomonas aeruginosa* infections. Drugs Context..

[117] Gurjar M (2015). Colistin for lung infection: an update. J Intensive Care..

[118] Sabuda DM, Laupland K, Pitout J, Dalton B, Rabin H, Louie T, Conly J (2008). Utilization of colistin for treatment of multidrug-resistant *Pseudomonas aeruginosa*. Can J Infect Dis Med Microbiol..

[119] Li H, Luo YF, Williams BJ, Blackwell TS, Xie CM (2012). Structure and function of OprD protein in *Pseudomonas aeruginosa*: from antibiotic resistance to novel therapies. Int J Med Microbiol..

[120] Cabot G, Ocampo-Sosa AA, Tubau F, Macia MD, Rodriguez C, Moya B (2011). Overexpression of *AmpC* and efflux pumps in *Pseudomonas aeruginosa* isolates from bloodstream infections: prevalence and impact on resistance in a Spanish multicenter study. Antimicrob Agents Chemother.

[121] Lee JY, Ko KS (2012). OprD mutations and inactivation, expression of efflux pumps and AmpC, and metallo-β-lactamases in carbapenem-resistant *Pseudomonas aeruginosa* isolates from South Korea. Int J Antimicrob Agents..

[122] Juan C, Torrens G, González-Nicolau M, Oliver A (2017). Diversity and regulation of intrinsic beta-lactamases from non-fermenting and other Gram-negative opportunistic pathogens. FEMS Microbiol Rev..

[123] Levy SB, Bonnie M (2004). Antibacterial resistance worldwide: Causes, challenges and responses. Nat Med.

[124] Law COK, Huang C, Pan Q, Lee J, Hao Q, Chan T-F (2019). A small RNA transforms the multidrug resistance of *Pseudomonas aeruginosa* to drug susceptibility. Mol Ther Nucleic Acids..

[125] Robicsek A, Strahilevitz J, Jacoby GA, Macielag M, Abbanat D, Park CH (2006). Fluoroquinolone-modifying enzyme: a new adaptation of a common aminoglycoside acetyltransferase. Nat Med..

[126] Ferreira I, Beisken S, Lueftinger L, Weinmaier T, Klein M, Bacher J (2020). Species identification and antibiotic resistance prediction by analysis of whole-genome sequence data by use of ARESdb: an analysis of isolates from the Unyvero Lower Respiratory Tract Infection Trial. J Clin Microbiol..

[127] Sarovich DS (2024). Supplemental ARDaP code..

