# Supplementary material for: Keeping up with the pathogens: improved antimicrobial resistance detection and prediction from Pseudomonas aeruginosa genomes
Source: Genome Med. 2024 Jun 7;16:78. doi: 10.1186/s13073-024-01346-z (PMC11157771; doi:10.1186/s13073-024-01346-z)
Supplement: Supplementary file 2 — Additional file 2: Fig. S1. Maximum likelihood phylogeny of ‘Validation Dataset’ isolates, their AMR profiles, and their disease origin. Fig. S2. Maximum likelihood phylogeny of all strains examined in this study. Fig. S3. Example clinician-friendly report produced by ARDaP. Table S1: Global Dataset strains, and their paired AMR and genomic data; Table S2: Validation Dataset strains generated in this study, and their AMR profiles. Table S3: Natural variants not associated with conferring AMR. Table S4: AMR rates in the Validation Dataset. Table S5: ARDaP performance across the Global Dataset when including intermediate resistance phenotype isolates. [file 13073_2024_1346_MOESM2_ESM.docx]

**Madden *et al*., 2024. Keeping up with the pathogens: Improved antimicrobial resistance detection and prediction from *Pseudomonas aeruginosa* genomes**


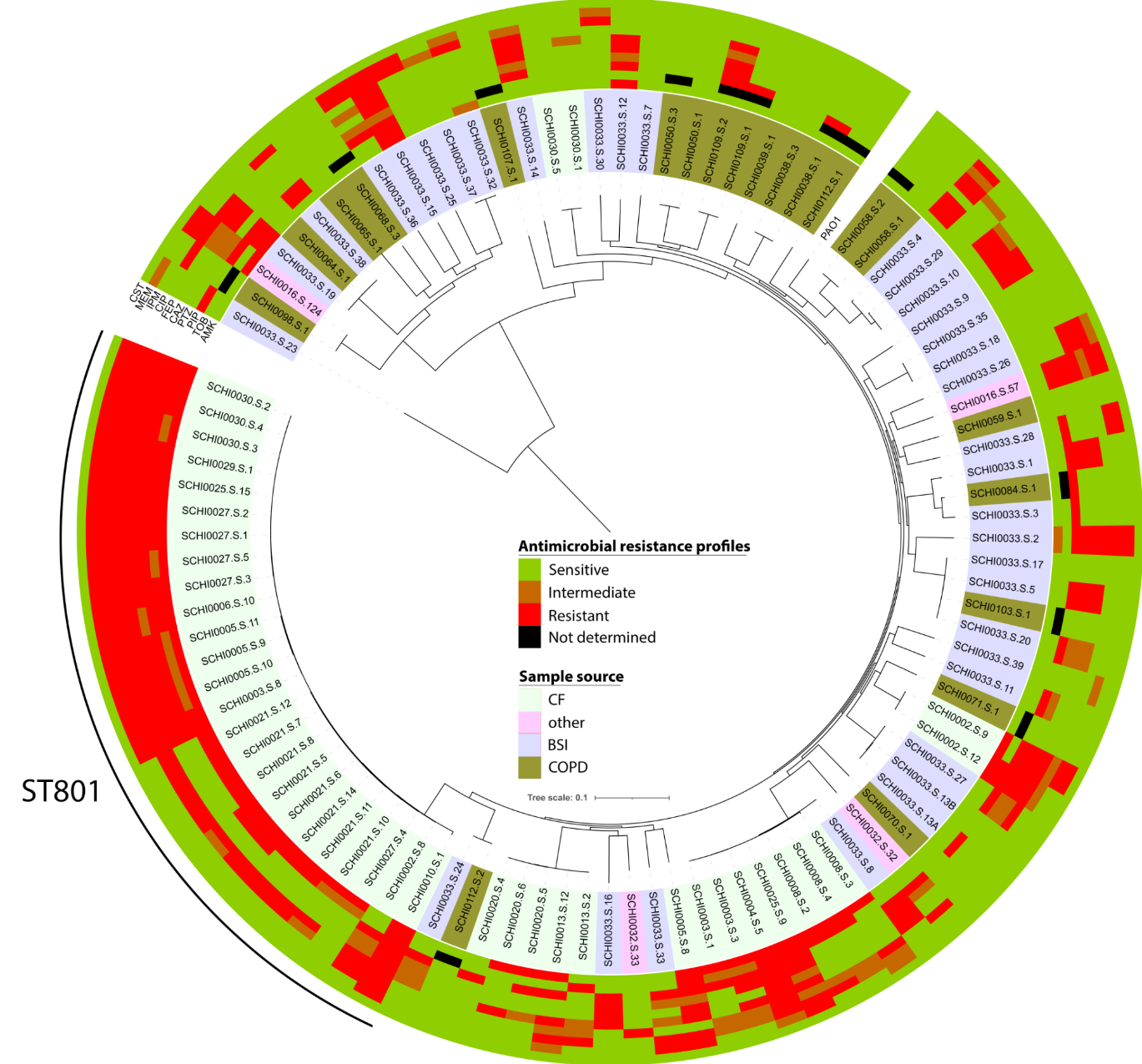


**Fig S1. Maximum likelihood phylogenomic analysis of the *Pseudomonas aeruginosa* ‘Validation Dataset’ generated in this study (*n*=102), along with their associated antimicrobial resistance profiles and disease origin.** *P. aeruginosa* PAO1 was used as the reference for alignment and single-nucleotide polymorphism identification. Variants were identified using the SPANDx v4.0.3 pipeline[1] with phylogenetic trees drawn with FastTree v2.1.10.[2] Shading represents isolate source and outer rings represent the antimicrobial resistance profile of the isolate. Abbreviations: AMK, amikacin; BSI, blood stream infection; CAZ, ceftazidime; CIP, ciprofloxacin; CST, colistin; CF, cystic fibrosis; COPD, chronic obstructive pulmonary disease; FEP, cefepime; IPM, imipenem; MEM, meropenem; PIP, piperacillin; ST, sequence type; TZP, piperacillin/tazobactam; TOB, tobramycin.


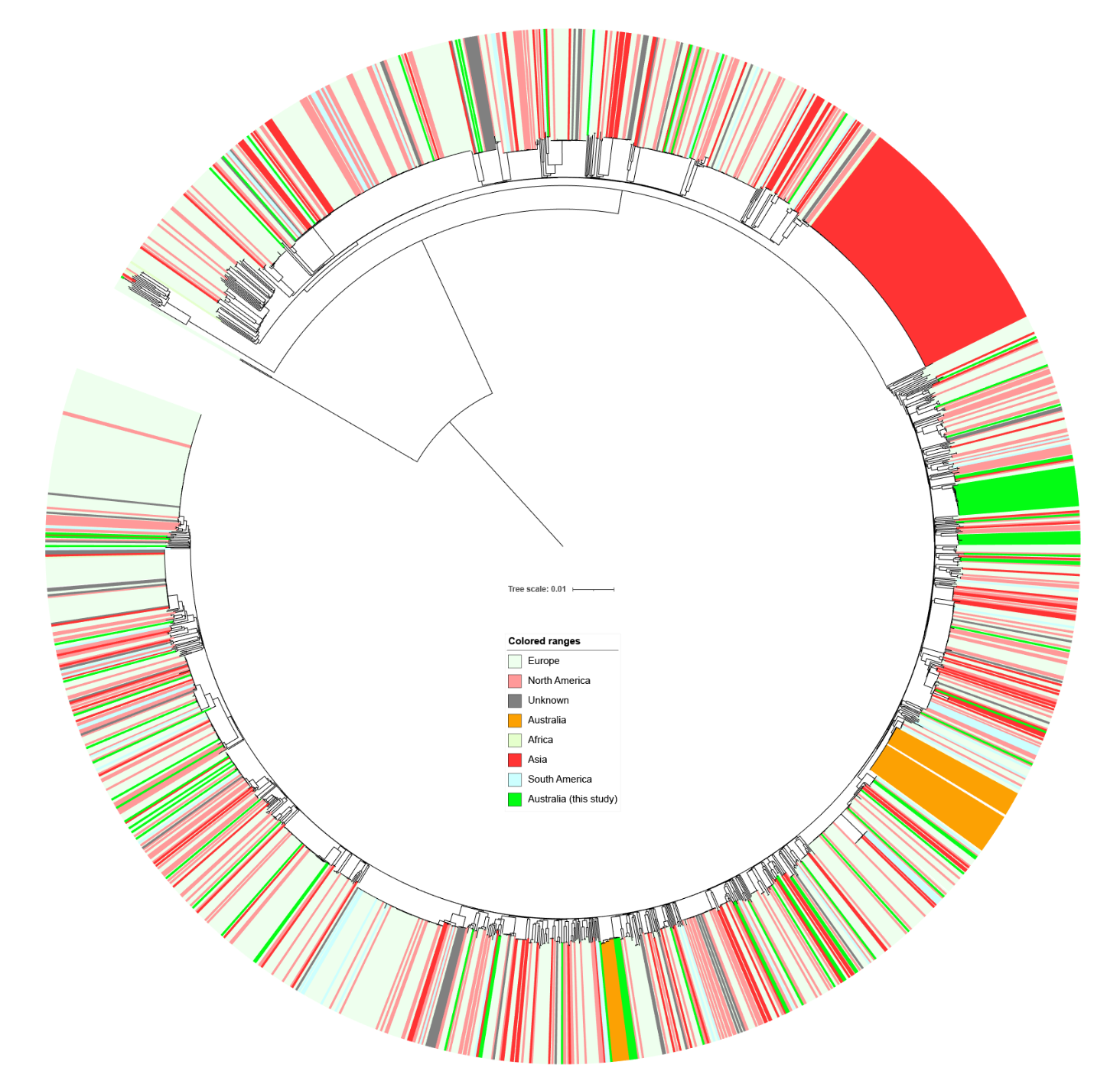


**Fig S2. Maximum likelihood phylogenomic analysis of all 1979 *Pseudomonas aeruginosa* strains examined in this study.** *P. aeruginosa* PAO1 was used as the reference genome for alignment and single-nucleotide polymorphism identification. Variants were identified using the SPANDx v4.0.3 pipeline[1], with phylogenetic trees drawn with FastTree v2.1.10[2]. Shading represents country of origin. The new Validation Dataset isolates generated in the current study (neon green) are distributed throughout the entire phylogeny, reflecting a panmictic population structure.


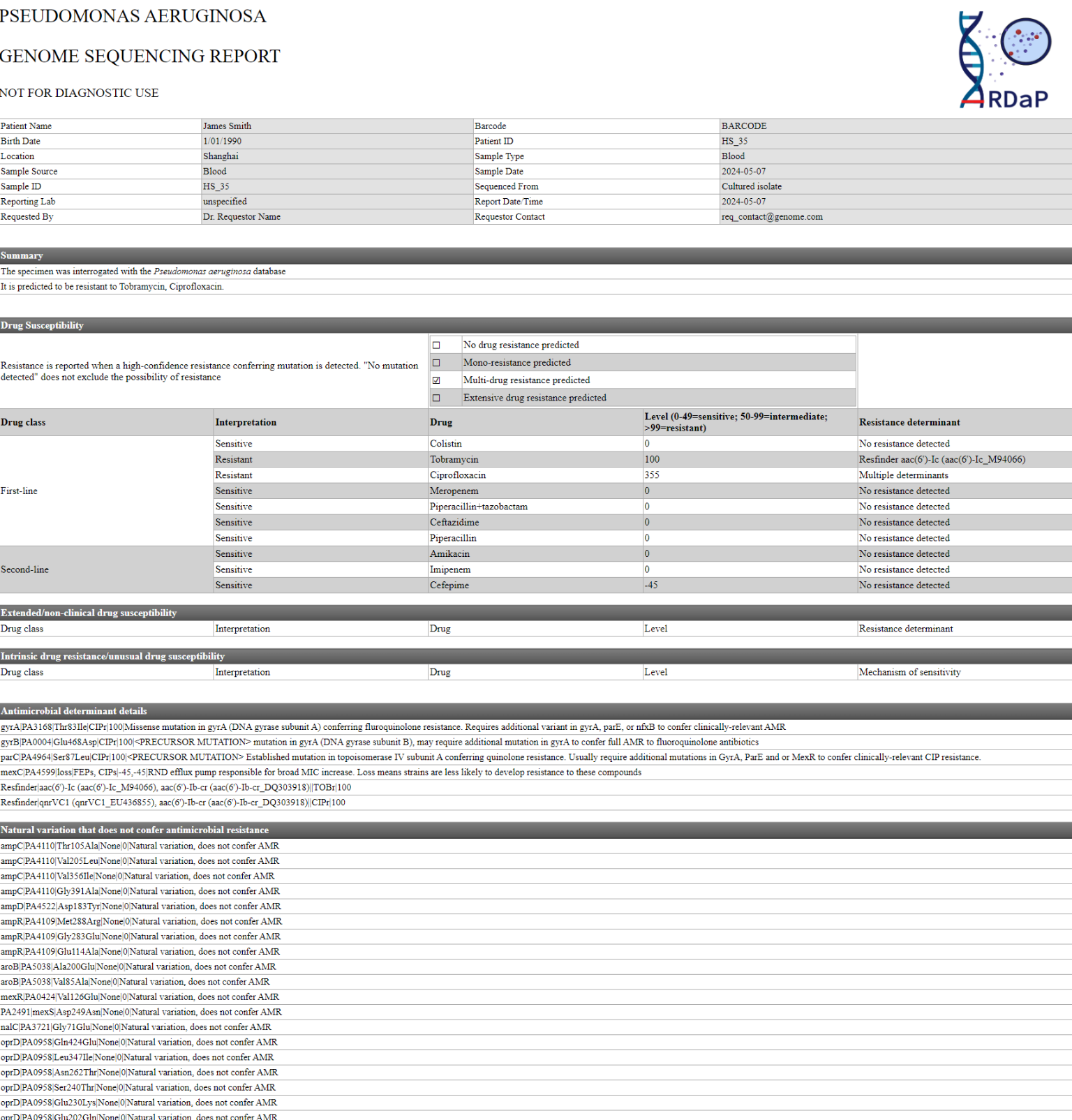


**Fig S3. Example clinician-friendly report produced by ARDaP for Chinese *Pseudomonas aeruginosa* strain HS_35.** This report summarises the predicted antimicrobial susceptibility profile, the individual drug susceptibility score, the drug susceptibility status (no resistance through to extensive drug resistance) for HS_35, and the corresponding antimicrobial resistance variants identified in this isolate.

**Table S1:** Summary of *Pseudomonas aeruginosa* Global Dataset strains with paired AMR and genomic data.

| **Study** | **No. strains^a^** | **Description** |
| --- | --- | --- |
| Kos *et al*., 2015 [3] | 385 | Diverse isolates collected from multiple geographical locations between 2003 and 2012 |
| van Belkum *et al*., 2015 [4] | 271^a,b^ | Diverse clinical isolates, mostly collected in Europe and USA, over a 25-year period |
| Cabot *et al*., 2016 [5] | 22 | MDR ST-175 isolates obtained from patients in 8 Spanish and 4 French hospitals, collected in 2008 and 2009 |
| Sherrard *et al*., 2017 [6] | 11 | MDR isolates collected from a cystic fibrosis patient in Queensland, Australia, between 2007 and 2014 |
| Ramanathan *et al*., 2017 [7] | 10 | Isolates collected from patients at a Malaysian hospital in 2009 and 2010 |
| Del Barrio-Tofiño *et al*., 2017 [8] | 41^a^ | MDR clinical isolates collected in Spain, 2015 |
| CDC-FDA Collection, 2018 [9] | 54 | MDR isolates from diverse clinical sources that represent the diversity of AMR phenotypes |
| Buhl *et al*., 2019 [10] | 45 | Clinical multidrug-resistant isolates obtained from two German hospitals during a four-year outbreak |
| Wardell *et al*., 2019 [11] | 39 | PAO1-derived isolates subjected to *in vitro* evolution for AMR development |
| Khaledi *et al*., 2020 [12] | 411 | Genetically diverse isolates with MDR obtained from European clinical cases. Temporal range unknown |
| Tsang *et al*., 2021 [13] | 102 | MDR isolates collected from patients admitted to Canadian hospitals between 2015 and 2018 |
| Cortes-Lara *et al.,* 2021 [14] | 289 | Diverse collection of isolates from Spanish hospitals. Collected in 2017 |
| Sun *et al*., 2023 [15] | 197 | Characterization of the genotypic features of an emerging ST316 sub lineage causing ear infections in Shanghai |
| **Total** | **1877** |  |

Abbreviations: AMR, antimicrobial-resistant; MDR, multidrug-resistant; ST, multi-locus sequence-type. ^a^Excludes genomes that were found to be of very low quality/coverage or missing from public databases. ^b^van Belkum *et al*., 2015 also included isolates from Kos *et al*., 2015. Strain numbers are representative of unique isolates only.

**Table S2:** *Pseudomonas aeruginosa* Validation Dataset strains generated in this study, and their corresponding antimicrobial susceptibility profiles.

| **Strain ID** | **Disease** | **Year of isolation** | **Antimicrobial sensitivity testing** | | | | | | | | | |
| --- | --- | --- | --- | --- | --- | --- | --- | --- | --- | --- | --- | --- |
|  |  |  | **AMK** | **TOB** | **PIP** | **TZP** | **CAZ** | **FEP** | **CIP** | **IPM** | **MEM** | **CST** |
| SCHI0002.S.8 | CF | 2017 | S | S | R | I | I | R | R | R | S | S |
| SCHI0002.S.9 | CF | 2017 | R | S | R | R | R | R | I | R | R | S |
| SCHI0002.S.12 | CF | 2018 | R | R | R | R | R | R | I | S | S | S |
| SCHI0003.S.1 | CF | 2017 | R | I | R | S | S | S | I | I | S | S |
| SCHI0003.S.3 | CF | 2017 | R | I | R | R | S | R | I | I | S | S |
| SCHI0003.S.8 | CF | 2017 | R | R | R | R | R | R | R | R | R | S |
| SCHI0004.S.5 | CF | 2017 | R | R | R | R | R | R | R | R | I | S |
| SCHI0005.S.8 | CF | 2017 | R | R | R | I | R | R | S | R | R | S |
| SCHI0005.S.9 | CF | 2017 | R | I | R | R | R | R | R | R | R | S |
| SCHI0005.S.10 | CF | 2017 | R | I | R | R | R | R | R | R | R | S |
| SCHI0005.S.11 | CF | 2017 | R | I | R | R | R | R | R | R | R | S |
| SCHI0006.S.10 | CF | 2017 | R | R | R | I | R | R | R | R | R | S |
| SCHI0008.S.2 | CF | 2018 | R | R | S | S | S | S | I | R | S | S |
| SCHI0008.S.3 | CF | 2018 | R | S | S | S | S | S | R | I | I | S |
| SCHI0008.S.4 | CF | 2018 | R | R | S | S | S | S | I | I | S | S |
| SCHI0010.S.1 | CF | 2018 | R | R | R | R | R | R | R | R | R | S |
| SCHI0013.S.2 | CF | 2018 | S | R | S | S | S | I | I | R | S | S |
| SCHI0013.S.12 | CF | 2018 | R | S | R | S | S | R | I | R | I | S |
| SCHI0016.S.57 | BE | 2017 | S | S | R | R | R | R | I | R | R | S |
| SCHI0016.S.124 | UTI | 2018 | R | S | R | I | I | I | R | R | R | S |
| SCHI0020.S.4 | CF | 2019 | S | S | R | S | S | S | S | S | S | S |
| SCHI0020.S.5 | CF | 2019 | R | S | R | S | S | I | S | S | S | S |
| SCHI0020.S.6 | CF | 2019 | R | S | S | S | S | R | S | S | S | S |
| SCHI0021.S.5 | CF | 2019 | R | R | R | S | S | R | I | S | S | S |
| SCHI0021.S.6 | CF | 2019 | R | R | R | S | S | R | R | S | S | S |
| SCHI0021.S.7 | CF | 2019 | R | R | S | S | S | R | R | S | S | S |
| SCHI0021.S.8 | CF | 2019 | R | R | S | S | S | R | R | S | S | S |
| SCHI0021.S.10 | CF | 2019 | R | I | S | S | S | R | I | S | S | S |
| SCHI0021.S.11 | CF | 2019 | R | R | S | S | S | R | R | S | S | S |
| SCHI0021.S.12 | CF | 2019 | R | R | S | S | S | R | R | S | S | S |
| SCHI0021.S.14 | CF | 2019 | R | R | S | S | S | R | R | S | S | S |
| SCHI0025.S.9 | CF | 2019 | R | R | R | R | I | R | I | R | R | S |
| SCHI0025.S.15 | CF | 2019 | R | R | R | R | R | R | R | R | R | S |
| SCHI0027.S.1 | CF | 2019 | R | R | R | R | R | R | R | R | R | S |
| SCHI0027.S.2 | CF | 2019 | R | R | R | R | R | R | R | R | R | S |
| SCHI0027.S.3 | CF | 2019 | R | R | R | R | R | R | R | R | R | S |
| SCHI0027.S.4 | CF | 2019 | R | I | S | S | S | S | S | S | S | S |
| SCHI0027.S.5 | CF | 2019 | R | I | R | R | R | R | R | R | R | S |
| SCHI0029.S.1 | CF | 2019 | R | R | R | R | R | R | R | R | R | S |
| SCHI0030.S.1 | CF | 2019 | S | S | S | S | S | I | S | S | S | S |
| SCHI0030.S.2 | CF | 2019 | R | R | R | R | R | R | R | R | R | S |
| SCHI0030.S.3 | CF | 2019 | R | I | R | R | R | R | R | R | R | S |
| SCHI0030.S.4 | CF | 2019 | R | R | R | R | R | R | R | R | R | S |
| SCHI0030.S.5 | CF | 2019 | S | S | S | S | S | S | S | S | S | S |
| SCHI0032.S.32 (AUS205) | Ulcer | 2008 | S | S | R | I | S | S | R | S | S | S |
| SCHI0032.S.33 (AUS134) | Ear Infection | 2008 | S | S | S | S | S | S | R | S | S | S |
| SCHI0033.S.1 | BSI | 2008 | S | S | R | R | R | R | S | S | S | S |
| SCHI0033.S.2 | BSI | 2009 | I | S | R | R | R | R | R | R | R | S |
| SCHI0033.S.3 | BSI | 2010 | S | S | R | S | S | S | S | S | S | S |
| SCHI0033.S.4 | BSI | 2010 | S | R | R | R | S | R | R | I | R | S |
| SCHI0033.S.5 | BSI | 2010 | S | S | R | R | R | R | S | S | S | S |
| SCHI0033.S.7 | BSI | 2010 | S | S | S | S | S | S | S | S | S | S |
| SCHI0033.S.8 | BSI | 2010 | S | S | R | R | R | R | S | S | S | S |
| SCHI0033.S.9 | BSI | 2010 | S | S | R | R | R | R | S | S | S | S |
| SCHI0033.S.10 | BSI | 2010 | S | S | R | R | R | I | S | S | S | S |
| SCHI0033.S.11 | BSI | 2012 | S | S | R | I | S | S | S | S | S | S |
| SCHI0033.S.12 | BSI | 2012 | R | S | R | I | R | R | S | S | S | S |
| SCHI0033.S.13A | BSI | 2012 | S | S | R | S | S | S | S | S | S | S |
| SCHI0033.S.13B | BSI | 2012 | S | S | S | S | S | S | S | S | S | S |
| SCHI0033.S.14 | BSI | 2008 | S | S | R | I | R | R | R | I | S | S |
| SCHI0033.S.15 | BSI | 2009 | R | R | R | I | R | R | R | R | R | S |
| SCHI0033.S.16 | BSI | 2009 | S | S | R | R | R | R | S | S | S | S |
| SCHI0033.S.17 | BSI | 2009 | S | S | S | S | S | S | S | S | S | S |
| SCHI0033.S.18 | BSI | 2010 | S | S | S | S | S | S | S | S | S | S |
| SCHI0033.S.19 | BSI | 2010 | R | R | R | S | R | R | R | S | S | S |
| SCHI0033.S.20 | BSI | 2010 | S | S | R | I | I | I | S | S | S | S |
| SCHI0033.S.23 | BSI | 2009 | S | S | R | S | S | S | S | S | I | S |
| SCHI0033.S.24 | BSI | 2009 | S | S | R | I | I | I | S | S | S | S |
| SCHI0033.S.25 | BSI | 2010 | S | S | S | S | S | S | S | R | R | S |
| SCHI0033.S.26 | BSI | 2010 | S | S | S | S | S | R | R | R | I | S |
| SCHI0033.S.27 | BSI | 2010 | S | S | R | I | I | R | S | S | S | S |
| SCHI0033.S.28 | BSI | 2010 | S | S | R | S | S | S | S | R | R | S |
| SCHI0033.S.29 | BSI | 2010 | S | S | S | S | S | S | I | S | S | S |
| SCHI0033.S.30 | BSI | 2010 | S | S | S | S | S | S | S | R | I | S |
| SCHI0033.S.32 | BSI | 2010 | I | S | S | S | S | S | S | R | I | S |
| SCHI0033.S.33 | BSI | 2010 | S | S | R | I | S | I | S | R | R | S |
| SCHI0033.S.35 | BSI | 2010 | S | S | S | S | S | S | S | S | S | S |
| SCHI0033.S.36 | BSI | 2012 | S | S | R | I | S | I | S | R | R | S |
| SCHI0033.S.37 | BSI | 2012 | S | S | S | S | S | S | S | I | S | S |
| SCHI0033.S.38 | BSI | 2012 | S | R | R | S | S | S | S | R | S | S |
| SCHI0033.S.39 | BSI | 2012 | S | S | S | S | S | S | S | I | S | S |
| SCHI0038.S.1 | COPD | 2020 | S | ND | R | S | S | S | S | S | S | S |
| SCHI0038.S.3 | COPD | 2020 | S | S | S | S | S | S | S | S | S | S |
| SCHI0039.S.1 | COPD | 2020 | S | S | S | S | S | S | S | S | S | S |
| SCHI0050.S.1 | COPD | 2020 | S | S | S | S | S | S | S | S | S | S |
| SCHI0050.S.3 | COPD | 2020 | S | ND | S | S | S | S | S | S | S | S |
| SCHI0058.S.1 | COPD | 2020 | S | S | S | S | S | S | S | S | S | S |
| SCHI0058.S.2 | COPD | 2021 | S | ND | S | S | S | S | S | S | S | S |
| SCHI0059.S.1 | COPD | 2020 | S | S | S | S | S | S | S | S | S | S |
| SCHI0064.S.1 | COPD | 2020 | S | S | S | S | S | S | S | S | S | S |
| SCHI0065.S.1 | COPD | 2020 | S | S | S | S | S | S | S | S | S | S |
| SCHI0068.S.3 | COPD | 2020 | S | ND | S | S | S | S | S | S | S | S |
| SCHI0070.S.1 | COPD | 2020 | S | S | S | S | S | S | S | S | S | S |
| SCHI0071.S.1 | COPD | 2020 | S | ND | S | S | S | S | S | S | S | S |
| SCHI0084.S.1 | COPD | 2021 | S | ND | R | S | S | S | S | S | S | S |
| SCHI0098.S.1 | COPD | 2021 | S | ND | S | S | S | S | R | S | S | S |
| SCHI0103.S.1 | COPD | 2021 | S | ND | S | S | S | S | S | S | S | S |
| SCHI0107.S.1 | COPD | 2022 | S | ND | S | S | S | S | S | S | S | S |
| SCHI0109.S.1 | COPD | 2021 | S | ND | R | S | S | S | S | S | S | S |
| SCHI0109.S.2 | COPD | 2022 | S | ND | R | R | R | I | R | S | S | S |
| SCHI0112.S.1 | COPD | 2021 | S | ND | S | S | S | S | S | S | S | S |
| SCHI0112.S.2 | COPD | 2022 | S | ND | S | S | S | S | S | S | S | S |

Abbreviations: AMK, amikacin; BE, bronchiectasis; BSI, blood stream infection; CAZ, ceftazidime; CF, cystic fibrosis; CIP, ciprofloxacin; COPD, chronic obstructive pulmonary disease; CST, colistin; FEP, cefepime; IPM, imipenem; MEM, meropenem; PIP, piperacillin; TOB, tobramycin; TZP, piperacillin/tazobactam; UTI, urinary tract infection.

Grey shading denotes intermediate (I) or resistant (R) phenotype for a given antibiotic. Non-grey-shaded cells denote a sensitive phenotype for a given antibiotic. ETESTs were used to determine minimum inhibitory concentrations for ciprofloxacin and meropenem. All other antibiotic resistance profiling was performed using disc diffusions.

**Table S3:** List of naturally occurring chromosomal determinants not associated with conferring antimicrobial resistance in *Pseudomonas aeruginosa*, and thus ignored by ARDaP.

| **Locus** | **Mutation** | **Reference** |
| --- | --- | --- |
| *PA0004* (*gyrB*) | Ser466Phe | [16] |
| *PA0090* (*clpV1*) | LOF | [17] |
| *PA0227* | LOF | [17] |
| *PA0262* | LOF | This study |
| *PA0295* | Ala155Thr | This study |
| *PA0373* (*ftsY*) | Glu127_Pro130dup | This study |
| *PA0424* (*mexR*) | LOF | [18] |
| *PA0424* (*mexR*) | Ile24fs | This study |
| *PA0424* (*mexR*) | Gly71Glu | [18] |
| *PA0424* (*mexR*) | Ala103Thr | This study |
| *PA0424* (*mexR*) | Ala108fs | This study |
| *PA0424* (*mexR*) | Ile111fs | This study |
| *PA0424* (*mexR*) | Val126Glu | [18, 19] |
| *PA0424* (*mexR*) | Glu153Gln | [18] |
| *PA0424* (*mexR*) | Ser209Arg | [18] |
| *PA0425* (*mexA*) | Gly108Thr | [20] |
| *PA0425* (*mexA*) | Gln183* | This study |
| *PA0425* (*mexA*) | Tyr197* | [11] |
| *PA0425* (*mexA*) | Cys360Gly | [11] |
| *PA0426* (*mexB*) | Val45Leu | [5] |
| *PA0426* (*mexB*) | Gln773* | This study |
| *PA0426* (*mexB*) | Met901fs | [11] |
| *PA0427* (*oprM*) | LOF | [21] |
| *PA0575* | LOF | This study |
| *PA0650* (*trpD*) | LOF | This study |
| *PA0799* | Ala275Thr | This study |
| *PA0946* | Arg287Trp | This study |
| *PA0958* (*oprD*) | Asp43Asn | This study |
| *PA0958* (*oprD*) | Ser57fs | This study |
| *PA0958* (*oprD*) | Ser57Glu | [22, 23] |
| *PA0958* (*oprD*) | Ser59Arg | [22, 23] |
| *PA0958* (*oprD*) | Ser59fs | This study |
| *PA0958* (*oprD*) | Thr103Ser | [23, 24] |
| *PA0958* (*oprD*) | Lys115Thr | [24] |
| *PA0958* (*oprD*) | Val127Leu | [22] |
| *PA0958* (*oprD*) | Val153Leu | This study |
| *PA0958* (*oprD*) | Phe170Leu | [23, 24] |
| *PA0958* (*oprD*) | Glu185Gln | [22-24] |
| *PA0958* (*oprD*) | Pro186fs | This study |
| *PA0958* (*oprD*) | Pro186Gly | [23, 24] |
| *PA0958* (*oprD*) | Thr187fs | This study |
| *PA0958* (*oprD*) | Val189del | This study |
| *PA0958* (*oprD*) | Val189Thr | [23, 24] |
| *PA0958* (*oprD*) | Glu202Gln | [23] |
| *PA0958* (*oprD*) | Ile210Ala | [23] |
| *PA0958* (*oprD*) | Glu230Lys | [22, 23] |
| *PA0958* (*oprD*) | Ser240Thr | [22, 23] |
| *PA0958* (*oprD*) | Asn262Thr | [22, 23] |
| *PA0958* (*oprD*) | Gly265Ser | [23] |
| *PA0958* (*oprD*) | Ala267Ser | [22] |
| *PA0958* (*oprD*) | Thr276Ala | [25] |
| *PA0958* (*oprD*) | Ala281fs | This study |
| *PA0958* (*oprD*) | Ala281Gly | [23] |
| *PA0958* (*oprD*) | Ala282fs | This study |
| *PA0958 (oprD)* | Ala293Pro | [24] |
| *PA0958* (*oprD*) | Lys296Gln | [23] |
| *PA0958* (*oprD*) | Gln301Glu | [23] |
| *PA0958* (*oprD*) | Arg310Gly | [22] |
| *PA0958* (*oprD*) | Ala315Gly | [22, 24] |
| *PA0958* (*oprD*) | Leu347Ile | This study |
| *PA0958* (*oprD*) | Val359Leu | [22] |
| *PA0958* (*oprD*) | Met372fs | [24, 26] |
| *PA0958* (*oprD*) | Met372Val | [22] |
| *PA0958* (*oprD*) | Asp374dup | This study |
| *PA0958* (*oprD*) | Asp374fs | This study |
| *PA0958* (*oprD*) | Asn375Ser | [22] |
| *PA0958* (*oprD*) | Asn376fs | This study |
| *PA0958* (*oprD*) | Asn376Ser | [22] |
| *PA0958* (*oprD*) | Val377fs | This study |
| *PA0958* (*oprD*) | Gly378fs | [22] |
| *PA0958* (*oprD*) | Lys380_Asn381del | This study |
| *PA0958* (*oprD*) | Lys380fs | This study |
| *PA0958* (*oprD*) | Lys380Tyr | [22] |
| *PA0958* (*oprD*) | Asn381fs | This study |
| *PA0958* (*oprD*) | Asn381His | This study |
| *PA0958* (*oprD*) | Tyr382_Gly383insAla | This study |
| *PA0958* (*oprD*) | Tyr382Gly | [22] |
| *PA0958* (*oprD*) | Gly383_Tyr384insLeu | This study |
| *PA0958* (*oprD*) | Asn407Ala | This study |
| *PA0958* (*oprD*) | His418Ala | [22] |
| *PA0958* (*oprD*) | Arg419Pro | [22] |
| *PA0958* (*oprD*) | Ala420Cys | [22] |
| *PA0958* (*oprD*) | Asn421Gln | [22] |
| *PA0958* (*oprD*) | Ala422_Asp423del | [22] |
| *PA0958* (*oprD*) | Ala422Arg | [22] |
| *PA0958* (*oprD*) | Asp423Arg | [22] |
| *PA0958* (*oprD*) | Gln424Glu | [22] |
| *PA0958* (*oprD*) | Gln424Pro | [22] |
| *PA0958* (*oprD*) | Gly425Ala | [24] |
| *PA0958* (*oprD*) | Glu426Arg | [22] |
| *PA0958* (*oprD*) | Gly427Arg | [22] |
| *PA0958* (*oprD*) | Asp428Arg | [22] |
| *PA0958* (*oprD*) | Gln429Pro | [22] |
| *PA0958* (*oprD*) | Asn430Glu | [22] |
| *PA0958* (*oprD*) | Glu431Arg | [22] |
| *PA0958* (*oprD*) | Tyr438fs | This study |
| *PA1017* (*paua*) | LOF | This study |
| *PA1045* | Lys322Arg | This study |
| *PA1101* (*fliF*) | LOF | [17] |
| *PA1167* | LOF | [17] |
| *PA1171* | LOF | [17] |
| *PA1180 (phoQ)* | LOF | [27] |
| *PA1180* (*phoQ*) | Val260Gly | [28] |
| *PA1195* | LOF | [17] |
| *PA1259* | LOF | [17] |
| *PA1316* | LOF | [17] |
| *PA1375* (*pdxB*) | Glu367Gly | This study |
| *PA1430* (*lasR*) | Gly191Ser | This study |
| *PA1487* | Arg185Ser | This study |
| *PA1549* (*copA2*) | LOF | [17] |
| *PA1549* (*copA2*) | Ala441fs | [11] |
| *PA1549* (*copA2*) | Ala442fs | [11] |
| *PA1611* | LOF | [17] |
| *PA1798* (*parS*) | LOF | This study |
| *PA1798* (*parS*) | Leu137Pro | [29] |
| *PA1798* (*parS*) | Ala138Thr | [29] |
| *PA1798* (*parS*) | His398Arg | [30] |
| *PA1799* (*parR*) | Met59Ile | This study |
| *PA1997* | Ile54Val | This study |
| *PA2020* | LOF | [29] |
| *PA2020* | Asn54_Lys55dup | This study |
| *PA2023* (*galU*) | LOF | [17] |
| *PA2064* (*pcoB*) | Gln59Lys | This study |
| *PA2119* | LOF | This study |
| *PA2128* (*cupA1*) | LOF | This study |
| *PA2152* | LOF | This study |
| *PA2198* | LOF | [17] |
| *PA2207* | LOF | [17] |
| *PA2252* | LOF | This study |
| *PA2326* | LOF | [17] |
| *PA2491 (mexS)* | LOF | [31] |
| *PA2491 (mexS)* | Arg48Cys | [32] |
| *PA2491* (*mexS*) | Ser124Arg | [31] |
| *PA2491 (mexS)* | Asp249Asn | [33] |
| *PA2492* (*mexT*) | Ala21fs | This study |
| *PA2492* (*mexT*) | Leu26Val | [34] |
| *PA2492* (*mexT*) | Arg28Ser | [35] |
| *PA2492* (*mexT*) | Ala39Val | [35] |
| *PA2492* (*mexT*) | Pro75fs | This study |
| *PA2492* (*mexT*) | Ala78fs | This study |
| *PA2492* (*mexT*) | Gln80fs | This study |
| *PA2492* (*mexT*) | Arg111fs | This study |
| *PA2492* (*mexT*) | Tyr164Asp | This study |
| *PA2492* (*mexT*) | Leu183fs | This study |
| *PA2492* (*mexT*) | Glu192fs | This study |
| *PA2492* (*mexT*) | Gly284Asp | This study |
| *PA2492* (*mexT*) | Ala293fs | This study |
| *PA2519* (*xylS*) | Thr242Pro | This study |
| *PA2571* | LOF | [17] |
| *PA2567* | Ile508Val | This study |
| *PA2638 (nuoB)* | LOF | This study |
| *PA2727* | Arg383Gln | This study |
| *PA2882* | Gln156His | This study |
| *PA2951* (*etfA*) | LOF | [17] |
| *PA2953* | LOF | [17] |
| *PA3015* | LOF | [17] |
| *PA3127* | LOF | [17] |
| *PA3168 (gyrA)* | Asp87Gly | [36, 37] |
| *PA3168* (*gyrA*) | Ala136Val | [37] |
| *PA3168* (*gyrA*) | Ala458Val | This study |
| *PA3178* | Ala115Val | This study |
| *PA3324* | LOF | [17] |
| *PA3351* (*flgM*) | LOF | [17] |
| *PA3418* (*ldh*) | LOF | This study |
| *PA3491* (*rnfC*) | Thr68Ser | This study |
| *PA3491* (*rnfC*) | His87Pro | [36] |
| *PA3491* (*rnfC*) | Cys198Arg | This study |
| *PA3491* (*rnfC*) | Ala639Val | [36] |
| *PA3491* (*rnfC*) | Gly652Arg | This study |
| *PA3491* (*rnfC*) | Ile726Thr | This study |
| *PA3491* (*rnfC*) | Ala751Pro | This study |
| *PA3491* (*rnfC*) | Ala759Thr | This study |
| *PA3574* | LOF | [17] |
| *PA3576* | LOF | [17] |
| *PA3574* (*nalD*) | Leu99fs | This study |
| *PA3574* (*nalD*) | Pro159Gln | This study |
| *PA3574* (*nalD*) | Asp187His | [38] |
| *PA3574 (nalD)* | Thr188Ala | [38, 39] |
| *PA3620 (mutS)* | LOF | [40] |
| *PA3622 (rpoS)* | LOF | This study |
| *PA3721* (*nalC*) | LOF | [11] |
| *PA3721* (*nalC*) | Thr50Pro | This study |
| *PA3721* (*nalC*) | Gly71Glu | [18, 34, 38, 41, 42] |
| *PA3721* (*nalC*) | Asp147Asn | [34] |
| *PA3721* (*nalC*) | Ala186Thr | [42] |
| *PA4020* (*mpl*) | Met38fs | [43] |
| *PA4020* (*mpl*) | Met297Val | This study |
| *PA4020* (*mpl*) | Arg322Gln | [44] |
| *PA4020 (mpl)* | LOF | [45] |
| *PA4020* (*mpl*) | Ter452Argext*? | [45] |
| *PA4025* | LOF | [17] |
| *PA4105* | LOF | This study |
| *PA4109* (*ampR*) | LOF | This study |
| *PA4109* (*ampR*) | Ala51Thr | [46] |
| *PA4109* (*ampR*) | Glu114Ala | [46] |
| *PA4109* (*ampR*) | Arg244Trp | [34] |
| *PA4109* (*ampR*) | Gly273Glu | [34] |
| *PA4109* (*ampR*) | Gly283Glu | [34, 46] |
| *PA4109* (*ampR*) | Met288Arg | [34, 46] |
| *PA4109* (*ampR*) | Glu292fs | This study |
| *PA4109* (*ampR*) | Ala293fs | This study |
| *PA4109* (*ampR*) | Arg294fs | This study |
| *PA4109* (*ampR*) | Arg296fs | This study |
| *PA4110* (*ampC*) | Thr21Ala | [47] |
| *PA4110* (*ampC*) | Gly27Asp | [47] |
| *PA4110* (*ampC*) | Gly27Val | [48] |
| *PA4110* (*ampC*) | Ala55Thr | [48] |
| *PA4110* (*ampC*) | Arg79Gln | [47, 48] |
| *PA4110* (*ampC*) | Ala97Val | [38, 47] |
| *PA4110* (*ampC*) | Thr105Ala | [47, 48] |
| *PA4110* (*ampC*) | Lys108Glu | [48] |
| *PA4110* (*ampC*) | Gln155Arg | [48] |
| *PA4110* (*ampC*) | Leu176Arg | [48] |
| *PA4110* (*ampC*) | Gly186Ser | [47] |
| *PA4110* (*ampC*) | Met201Leu | [48] |
| *PA4110* (*ampC*) | Val205Leu | [48] |
| *PA4110* (*ampC*) | Val356Ile | [48] |
| *PA4110* (*ampC*) | Gly391Ala | [48] |
| *PA4120* | Ser158fs | This study |
| *PA4120* | Gly159fs | This study |
| *PA4218 (ampP)* | Met87Ile | [44] |
| *PA4218 (ampP)* | Arg171Cys | [44] |
| *PA4218 (ampP)* | Thr172Ala | [44] |
| *PA4266* (*fusA1*) | Ala21Val | [11] |
| *PA4266* (*fusA1*) | Val93Ala | [49] |
| *PA4266* (*fusA1*) | Ile186Val | [49] |
| *PA4293 (pprA)* | LOF | [6] |
| *PA4310 (pctB)* | LOF | This study |
| *PA4315 (mvtA)* | LOF | [33] |
| *PA4334* | LOF | This study |
| *PA4375* | Gly930Glu | This study |
| *PA4418* (*ftsI*) | Pro215Leu | [50, 51] |
| *PA4418* (*ftsI*) | Gly216Ser | [50] |
| *PA4490* | LOF | [17] |
| *PA4521 (ampE)* | Ser69Pro | [44] |
| *PA4522* (*ampD*) | Asp28Gly | [52] |
| *PA4522* (*ampD*) | Gly46Ser | This study |
| *PA4522* (*ampD*) | His98Arg | This study |
| *PA4522* (*ampD*) | Ala136Val | [53] |
| *PA4522* (*ampD*) | Glu148Ala | [44] |
| *PA4522* (*ampD*) | Ser175Leu | [53] |
| *PA4522* (*ampD*) | Asp183Tyr | [44] |
| *PA4539* | LOF | This study |
| *PA4556* (*pilE*) | Leu121fs | This study |
| *PA4598* (*mexD*) | LOF | This study |
| *PA4599* (*mexC*) | -67C>T | This study |
| *PA2494* (*mexF*) | LOF | This study |
| *PA4600* (*nfxB*) | Arg21His | [54] |
| *PA4600* (*nfxB*) | Asp56Gly | [55] |
| *PA4600* (*nfxB*) | Arg82Leu | [56] |
| *PA4600 (nfxB)* | LOF | [37] |
| *PA4622* | Ile380Val | This study |
| *PA4777* (*pmrB*) | Val15Ile | [57] |
| *PA4777* (*pmrB*) | Ala67Thr | [57] |
| *PA4777* (*pmrB*) | Asp70Asn | [57] |
| *PA4777* (*pmrB*) | Met292Ile | [58, 59] |
| *PA4777* (*pmrB*) | His340Arg | [57] |
| *PA4777* (*pmrB*) | Thr343Ala | [57] |
| *PA4777* (*pmrB*) | Tyr345His | [60] |
| *PA4778* (*cueR*) | Gly57Ala | This study |
| *PA4781* | LOF | [17] |
| *PA4898 (opdK)* | LOF | This study |
| *PA4964 (parC)* | Gln405Arg | This study |
| *PA4964* (*parC*) | Val646Leu | This study |
| *PA4967* (*parE*) | Met437Ile | This study |
| *PA4967* (*parE*) | Ala473Val | [61-63] |
| *PA4967* (*parE*) | Asp533Glu | This study |
| *PA5001* | LOF | [17, 64] |
| *PA5003* | LOF | [17, 64] |
| *PA5003* | Arg268* | This study |
| *PA5038* (*aroB*) | Val85Ala | [5] |
| *PA5038* (*aroB*) | Ala200Glu | [5] |
| *PA5038* (*aroB*) | Thr211fs | This study |
| *PA5087* | Leu169Val | This study |
| *PA5114* | LOF | [17] |
| *PA5192 (pckA*) | LOF | [65] |
| *PA5192* (*pckA*) | Ala461Thr | This study |
| *PA5238* | Val85fs | This study |
| *PA5253* (*algP*) | LOF | This study |
| *PA5471* (*armZ*) | Upregulation | [29] |
| *PA5485* (*ampDh2*) | -12_-11delTT | This study |
| *PA5528* | LOF | [66] |

Abbreviations: fs, frameshift; LOF, loss of function; *, stop codon. NB. Natural variation, including LOF mutations, does not exclude a gene as having a role in conferring antimicrobial resistance.

**Table S4:** Antimicrobial resistance rates across the Validation Dataset (*n*=102).

| **Antibiotic Class** | **Antibiotic** | **Resistant (%)** | **Intermediate (%)** | **Sensitive (%)** |
| --- | --- | --- | --- | --- |
| Carbapenems | MEM | 27 (26) | 7 (7) | 68 (67) |
|  | IPM | 35 (34) | 8 (8) | 59 (58) |
| Polymyxins | CST | 0 (0) | 0 (0) | 102 (100) |
| Fluoroquinolones | CIP | 35 (34) | 13 (13) | 54 (53) |
| Cephalosporins | FEP | 46 (45) | 10 (10) | 46 (45) |
|  | CAZ | 32 (31) | 6 (6) | 64 (63) |
| Penicillins | TZP | 29 (28) | 14 (14) | 59 (58) |
|  | PIP | 58 (57) | 0 (0) | 44 (43) |
| Aminoglycosides | TOB | 28 (31) | 9 (10) | 52 (58) |
|  | AMK | 41 (40) | 2 (2) | 59 (55) |

MEM, meropenem; IPM, imipenem; CST, colistin; CIP, ciprofloxacin; FEP, cefepime; CAZ, ceftazidime, TZP, piperacillin/tazobactam; PIP, piperacillin; TOB, tobramycin; AMK, amikacin

**Table S5:** Assessment of ARDaP performance across the Global Dataset when including isolates with an intermediate resistance phenotype.

| **Antibiotic Class** | **Antibiotic** | **True Positive** | **True Negative** | **False Positive** | **False Negative** | **Specificity** | **Sensitivity** | **bACC** |
| --- | --- | --- | --- | --- | --- | --- | --- | --- |
| Carbapenems | MEM | 563 | 991 | 133 | 188 | 0.88 | 0.75 | 0.82 |
|  | IPM | 144 | 405 | 14 | 84 | 0.96 | 0.63 | 0.79 |
| Polymyxins | CST | 10 | 933 | 0 | 38 | 1.00 | 0.21 | 0.60 |
| Fluoroquinolones | CIP | 627 | 430 | 47 | 80 | 0.85 | 0.88 | 0.86 |
| Cephalosporins | FEP | 127 | 617 | 33 | 96 | 0.90 | 0.54 | 0.72 |
|  | CAZ | 255 | 690 | 43 | 119 | 0.90 | 0.63 | 0.77 |
| Penicillins | TZP | 112 | 504 | 22 | 81 | 0.93 | 0.52 | 0.72 |
|  | PIP | 154 | 255 | 17 | 41 | 0.91 | 0.77 | 0.84 |
| Aminoglycosides | TOB | 271 | 675 | 20 | 23 | 0.97 | 0.90 | 0.93 |
|  | AMK | 143 | 1158 | 39 | 48 | 0.94 | 0.72 | 0.83 |

Abbreviations: MEM, meropenem; IPM, imipenem; CST, colistin; CIP, ciprofloxacin; FEP, cefepime; CAZ, ceftazidime, TZP, piperacillin/tazobactam; PIP, piperacillin; TOB, tobramycin; AMK, amikacin; bACC, balanced accuracy.

**References**

1. Sarovich DS, Price EP: **SPANDx: a genomics pipeline for comparative analysis of large haploid whole genome re-sequencing datasets**. *BMC research notes* 2014, **7**:618.

2. Price MN, Dehal PS, Arkin AP: **FastTree 2 – approximately maximum-likelihood trees for large alignments**. *PLoS One* 2010, **5**(3):e9490.

3. Kos VN, Déraspe M, McLaughlin RE, Whiteaker JD, Roy PH, Alm RA, Corbeil J, Gardner H: **The resistome of *Pseudomonas aeruginosa* in relationship to phenotypic susceptibility**. *Antimicrobial agents and chemotherapy* 2015, **59**(1):427-436.

4. van Belkum A, Soriaga LB, LaFave MC, Akella S, Veyrieras J-B, Barbu EM, Shortridge D, Blanc B, Hannum G, Zambardi G *et al*: **Phylogenetic distribution of CRISPR-Cas systems in antibiotic-resistant *Pseudomonas aeruginosa***. *mBio* 2015, **6**(6):e01796-01715.

5. Cabot G, López-Causapé C, Ocampo-Sosa AA, Sommer LM, Domínguez MÁ, Zamorano L, Juan C, Tubau F, Rodríguez C, Moyà B *et al*: **Deciphering the resistome of the widespread *Pseudomonas aeruginosa* sequence type 175 international high-risk clone through whole-genome sequencing**. *Antimicrobial agents and chemotherapy* 2016, **60**(12):7415-7423.

6. Sherrard LJ, Tai AS, Wee BA, Ramsay KA, Kidd TJ, Ben Zakour NL, Whiley DM, Beatson SA, Bell SC: **Within-host whole genome analysis of an antibiotic resistant *Pseudomonas aeruginosa* strain sub-type in cystic fibrosis**. *PLoS One* 2017, **12**(3):e0172179.

7. Ramanathan B, Jindal HM, Le CF, Gudimella R, Anwar A, Razali R, Poole-Johnson J, Manikam R, Sekaran SD: **Next generation sequencing reveals the antibiotic resistant variants in the genome of *Pseudomonas aeruginosa***. *PLoS One* 2017, **12**(8):e0182524.

8. del Barrio-Tofiño E, López-Causapé C, Cabot G, Rivera A, Benito N, Segura C, Montero MM, Sorlí L, Tubau F, Gómez-Zorrilla S *et al*: **Genomics and susceptibility profiles of extensively drug-resistant *Pseudomonas aeruginosa* isolates from Spain**. *Antimicrobial Agents and Chemotherapy* 2017, **61**(11):e01589-01517.

9. **CDC & FDA Antibiotic Resistance Isolate Bank** [<https://wwwn.cdc.gov/ARIsolateBank/Panel/PanelDetail?ID=12>, accessed 18Jan22]]

10. Buhl M, Kästle C, Geyer A, Autenrieth IB, Peter S, Willmann M: **Molecular evolution of extensively drug-resistant (XDR) *Pseudomonas aeruginosa* strains from patients and hospital environment in a prolonged outbreak**. *Front Microbiol* 2019, **10**:1742.

11. Wardell SJT, Rehman A, Martin LW, Winstanley C, Patrick WM, Lamont IL: **A large-scale whole-genome comparison shows that experimental evolution in response to antibiotics predicts changes in naturally evolved clinical *Pseudomonas aeruginosa***. *Antimicrob Agents Chemother* 2019, **63**(12):e01619-01619.

12. Khaledi A, Weimann A, Schniederjans M, Asgari E, Kuo T-H, Oliver A, Cabot G, Kola A, Gastmeier P, Hogardt M *et al*: **Predicting antimicrobial resistance in *Pseudomonas aeruginosa* with machine learning-enabled molecular diagnostics**. *EMBO Mol Med* 2020, **12**(3):e10264.

13. Tsang KK, Maguire F, Zubyk HL, Chou S, Edalatmand A, Wright GD, Beiko RG, McArthur AG: **Identifying novel β-lactamase substrate activity through *in silico* prediction of antimicrobial resistance**. *Microb Genom* 2021, **7**(1):mgen000500.

14. Cortes-Lara S, Barrio-Tofiño ED, López-Causapé C, Oliver A, Gemara-Seimc Reipi Pseudomonas study Group: **Predicting *Pseudomonas aeruginosa* susceptibility phenotypes from whole genome sequence resistome analysis**. *Clinical microbiology and infection : the official publication of the European Society of Clinical Microbiology and Infectious Diseases* 2021, **27**(11):1631-1637.

15. Sun Z, Yang F, Ji J, Cao W, Liu C, Ding B, Xu X: **Dissecting the genotypic features of a fluoroquinolone-resistant *Pseudomonas aeruginosa* ST316 sublineage causing ear infections in Shanghai, China**. *Microbial genomics* 2023, **9**(4).

16. Bruchmann S, Dötsch A, Nouri B, Chaberny IF, Häussler S: **Quantitative contributions of target alteration and decreased drug accumulation to *Pseudomonas aeruginosa* fluoroquinolone resistance**. *Antimicrobial agents and chemotherapy* 2013, **57**(3):1361-1368.

17. Dötsch A, Becker T, Pommerenke C, Magnowska Z, Jänsch L, Häussler S: **Genomewide identification of genetic determinants of antimicrobial drug resistance in *Pseudomonas aeruginosa***. *Antimicrobial agents and chemotherapy* 2009, **53**(6):2522-2531.

18. Llanes C, Hocquet D, Vogne C, Benali-Baitich D, Neuwirth C, Plésiat P: **Clinical strains of *Pseudomonas aeruginosa* overproducing MexAB-OprM and MexXY efflux pumps simultaneously**. *Antimicrob Agents Chemother* 2004, **48**(5):1797-1802.

19. Nguyen KV, Nguyen TV, Nguyen HTT, Le DV: **Mutations in the *gyrA*, *parC*, and *mexR* genes provide functional insights into the fluoroquinolone-resistant *Pseudomonas aeruginosa* isolated in Vietnam**. *Infect Drug Resist* 2018, **11**:275-282.

20. Chalhoub H, Sáenz Y, Nichols WW, Tulkens PM, Van Bambeke F: **Loss of activity of ceftazidime-avibactam due to MexAB-OprM efflux and overproduction of AmpC cephalosporinase in *Pseudomonas aeruginosa* isolated from patients suffering from cystic fibrosis**. *Int J Antimicrob Agents* 2018, **52**(5):697-701.

21. Wang H, Meng J, Jia M, Ma X, He G, Yu J, Wang R, Bai H, Hou Z, Luo X: **oprM as a new target for reversion of multidrug resistance in Pseudomonas aeruginosa by antisense phosphorothioate oligodeoxynucleotides**. *FEMS Immunol Med Microbiol* 2010, **60**(3):275-282.

22. González-Vázquez MC, Rocha-Gracia RDC, Carabarín-Lima A, Bello-López E, Huerta-Romano F, Martínez-Laguna Y, Lozano-Zarain P: **Location of OprD porin in *Pseudomonas aeruginosa* clinical isolates**. *APMIS* 2021, **129**(4):213-224.

23. Shu JC, Kuo AJ, Su LH, Liu TP, Lee MH, Su IN, Wu TL: **Development of carbapenem resistance in *Pseudomonas aeruginosa* is associated with OprD polymorphisms, particularly the amino acid substitution at codon 170**. *J Antimicrob Chemother* 2017, **72**(9):2489-2495.

24. Rodríguez-Martínez JM, Poirel L, Nordmann P: **Molecular epidemiology and mechanisms of carbapenem resistance in *Pseudomonas aeruginosa***. *Antimicrobial Agents and Chemotherapy* 2009, **53**(11):4783-4788.

25. Ocampo-Sosa AA, Cabot G, Rodríguez C, Roman E, Tubau F, Macia MD, Moya B, Zamorano L, Suárez C, Peña C *et al*: **Alterations of OprD in carbapenem-intermediate and -susceptible strains of *Pseudomonas aeruginosa* isolated from patients with bacteremia in a Spanish multicenter study**. *Antimicrob Agents Chemother* 2012, **56**(4):1703-1713.

26. Epp SF, Köhler T, Plésiat P, Michéa-Hamzehpour M, Frey J, Pechère JC: **C-terminal region of *Pseudomonas aeruginosa* outer membrane porin OprD modulates susceptibility to meropenem**. *Antimicrob Agents Chemother* 2001, **45**(6):1780-1787.

27. Lu L, Akerbladh L, Ahmad S, Konda V, Cao S, Vocat A, Maes L, Cole ST, Hughes D, Larhed M *et al*: **Synthesis and In Vitro Biological Evaluation of Quinolinyl Pyrimidines Targeting Type II NADH-Dehydrogenase (NDH-2)**. *ACS Infect Dis* 2022, **8**(3):482-498.

28. Barrow K, Kwon DH: **Alterations in two-component regulatory systems of *phoPQ* and *pmrAB* are associated with polymyxin B resistance in clinical isolates of *Pseudomonas aeruginosa***. *Antimicrob Agents Chemother* 2009, **53**(12):5150-5154.

29. Guénard S, Muller C, Monlezun L, Benas P, Broutin I, Jeannot K, Plésiat P: **Multiple mutations lead to MexXY-OprM-dependent aminoglycoside resistance in clinical strains of *Pseudomonas aeruginosa***. *Antimicrobial agents and chemotherapy* 2014, **58**(1):221-228.

30. Lee JY, Chung ES, Na IY, Kim H, Shin D, Ko KS: **Development of colistin resistance in *pmrA*-, *phoP*-, *parR*- and *cprR*-inactivated mutants of *Pseudomonas aeruginosa***. *J Antimicrob Chemother* 2014, **69**(11):2966-2971.

31. Sobel ML, Neshat S, Poole K: **Mutations in *PA2491* (*mexS*) promote MexT-dependent *mexEF-oprN* expression and multidrug resistance in a clinical strain of *Pseudomonas aeruginosa***. *J Bacteriol* 2005, **187**(4):1246-1253.

32. Petitjean M, Martak D, Silvant A, Bertrand X, Valot B, Hocquet D: **Genomic characterization of a local epidemic Pseudomonas aeruginosa reveals specific features of the widespread clone ST395**. *Microbial Genomics* 2017, **3**(10).

33. Llanes C, Köhler T, Patry I, Dehecq B, van Delden C, Plésiat P: **Role of the MexEF-OprN efflux system in low-level resistance of *Pseudomonas aeruginosa* to ciprofloxacin**. *Antimicrobial agents and chemotherapy* 2011, **55**(12):5676-5684.

34. Quale J, Bratu S, Gupta J, Landman D: **Interplay of efflux system, *ampC*, and *oprD* expression in carbapenem resistance of *Pseudomonas aeruginosa* clinical isolates**. *Antimicrobial Agents and Chemotherapy* 2006, **50**(5):1633-1641.

35. LoVullo ED, Schweizer HP: ***Pseudomonas aeruginosa mexT* is an indicator of PAO1 strain integrity**. *J Med Microbiol* 2020, **69**(1):139-145.

36. Rehman A, Jeukens J, Levesque RC, Lamont IL: **Gene-gene interactions dictate ciprofloxacin resistance in *Pseudomonas aeruginosa* and facilitate prediction of resistance phenotype from genome sequence data**. *Antimicrobial agents and chemotherapy* 2021, **65**(7):e0269620.

37. Higgins PG, Fluit AC, Milatovic D, Verhoef J, Schmitz FJ: **Mutations in GyrA, ParC, MexR and NfxB in clinical isolates of *Pseudomonas aeruginosa***. *International journal of antimicrobial agents* 2003, **21**(5):409-413.

38. Tomás M, Doumith M, Warner M, Turton JF, Beceiro A, Bou G, Livermore DM, Woodford N: **Efflux pumps, OprD porin, AmpC beta-lactamase, and multiresistance in *Pseudomonas aeruginosa* isolates from cystic fibrosis patients**. *Antimicrobial agents and chemotherapy* 2010, **54**(5):2219-2224.

39. Quale J, Bratu S, Gupta J, Landman D: **Interplay of efflux system, *ampC*, and *oprD* expression in carbapenem resistance of *Pseudomonas aeruginosa* clinical isolates**. *Antimicrobial agents and chemotherapy* 2006, **50**(5):1633-1641.

40. Oliver A, Baquero F, Blázquez J: **The mismatch repair system (*mutS*, *mutL* and *uvrD* genes) in *Pseudomonas aeruginosa*: molecular characterization of naturally occurring mutants**. *Mol Microbiol* 2002, **43**(6):1641-1650.

41. Díaz-Ríos C, Hernández M, Abad D, Álvarez-Montes L, Varsaki A, Iturbe D, Calvo J, Ocampo-Sosa AA: **New sequence type ST3449 in multidrug-resistant *Pseudomonas aeruginosa* isolates from a cystic fibrosis patient**. *Antibiotics (Basel)* 2021, **10**(5).

42. Braz VS, Furlan JP, Fernandes AF, Stehling EG: **Mutations in NalC induce MexAB-OprM overexpression resulting in high level of aztreonam resistance in environmental isolates of *Pseudomonas aeruginosa***. *FEMS Microbiol Lett* 2016, **363**(16).

43. Wang K, Chen YQ, Salido MM, Kohli GS, Kong JL, Liang HJ, Yao ZT, Xie YT, Wu HY, Cai SQ *et al*: **The rapid *in vivo* evolution of *Pseudomonas aeruginosa* in ventilator-associated pneumonia patients leads to attenuated virulence**. *Open Biol* 2017, **7**(9).

44. Li Z, Cai Z, Cai Z, Zhang Y, Fu T, Jin Y, Cheng Z, Jin S, Wu W, Yang L *et al*: **Molecular genetic analysis of an XDR *Pseudomonas aeruginosa* ST664 clone carrying multiple conjugal plasmids**. *J Antimicrob Chemother* 2020, **75**(6):1443-1452.

45. Tsutsumi Y, Tomita H, Tanimoto K: **Identification of novel genes responsible for overexpression of *ampC* in *Pseudomonas aeruginosa* PAO1**. *Antimicrobial agents and chemotherapy* 2013, **57**(12):5987-5993.

46. Balasubramanian D, Kumari H, Mathee K: ***Pseudomonas aeruginosa* AmpR: an acute-chronic switch regulator**. *Pathog Dis* 2015, **73**(2):1-14.

47. Tam VH, Schilling AN, LaRocco MT, Gentry LO, Lolans K, Quinn JP, Garey KW: **Prevalence of AmpC over-expression in bloodstream isolates of *Pseudomonas aeruginosa***. *Clin Microbiol Infect* 2007, **13**(4):413-418.

48. Berrazeg M, Jeannot K, Ntsogo Enguéné VY, Broutin I, Loeffert S, Fournier D, Plésiat P: **Mutations in β-Lactamase AmpC increase resistance of *Pseudomonas aeruginosa* isolates to antipseudomonal cephalosporins**. *Antimicrobial Agents and Chemotherapy* 2015, **59**(10):6248-6255.

49. Bolard A, Plésiat P, Jeannot K: **Mutations in gene *fusA1* as a novel mechanism of aminoglycoside resistance in clinical strains of *Pseudomonas aeruginosa***. *Antimicrobial agents and chemotherapy* 2018, **62**(2).

50. López-Causapé C, Sommer LM, Cabot G, Rubio R, Ocampo-Sosa AA, Johansen HK, Figuerola J, Cantón R, Kidd TJ, Molin S *et al*: **Evolution of the *Pseudomonas aeruginosa* mutational resistome in an international cystic fibrosis clone**. *Sci Rep* 2017, **7**(1):5555.

51. McLean K, Lee D, Holmes EA, Penewit K, Waalkes A, Ren M, Lee SA, Gasper J, Manoil C, Salipante SJ: **Genomic analysis identifies novel *Pseudomonas aeruginosa* resistance genes under selection during inhaled aztreonam therapy *in vivo***. *Antimicrob Agents Chemother* 2019, **63**(9).

52. Tueffers L, Barbosa C, Bobis I, Schubert S, Höppner M, Rühlemann M, Franke A, Rosenstiel P, Friedrichs A, Krenz-Weinreich A *et al*: ***Pseudomonas aeruginosa* populations in the cystic fibrosis lung lose susceptibility to newly applied β-lactams within 3 days**. *J Antimicrob Chemother* 2019, **74**(10):2916-2925.

53. Langaee TY, Gagnon L, Huletsky A: **Inactivation of the *ampD* gene in *Pseudomonas aeruginosa* leads to moderate-basal-level and hyperinducible AmpC β-lactamase expression**. *Antimicrob Agents Chemother* 2000, **44**(3):583-589.

54. Jeannot K, Elsen S, Köhler T, Attree I, van Delden C, Plésiat P: **Resistance and virulence of *Pseudomonas aeruginosa* clinical strains overproducing the MexCD-OprJ efflux pump**. *Antimicrob Agents Chemother* 2008, **52**(7):2455-2462.

55. Campo Esquisabel AB, Rodríguez MC, Campo-Sosa AO, Rodríguez C, Martínez-Martínez L: **Mechanisms of resistance in clinical isolates of *Pseudomonas aeruginosa* less susceptible to cefepime than to ceftazidime**. *Clin Microbiol Infect* 2011, **17**(12):1817-1822.

56. Jalal S, Ciofu O, Hoiby N, Gotoh N, Wretlind B: **Molecular mechanisms of fluoroquinolone resistance in *Pseudomonas aeruginosa* isolates from cystic fibrosis patients**. *Antimicrob Agents Chemother* 2000, **44**(3):710-712.

57. Lee JY, Ko KS: **Mutations and expression of PmrAB and PhoPQ related with colistin resistance in *Pseudomonas aeruginosa* clinical isolates**. *Diagn Microbiol Infect Dis* 2014, **78**(3):271-276.

58. Abraham N, Kwon DH: **A single amino acid substitution in PmrB is associated with polymyxin B resistance in clinical isolate of *Pseudomonas aeruginosa***. *FEMS Microbiol Lett* 2009, **298**(2):249-254.

59. Moskowitz SM, Brannon MK, Dasgupta N, Pier M, Sgambati N, Miller AK, Selgrade SE, Miller SI, Denton M, Conway SP *et al*: **PmrB mutations promote polymyxin resistance of *Pseudomonas aeruginosa* isolated from colistin-treated cystic fibrosis patients**. *Antimicrobial agents and chemotherapy* 2012, **56**(2):1019-1030.

60. Owusu-Anim D, Kwon DH: **Differential role of two-component regulatory cystems (*phoPQ* and *pmrAB*) in polymyxin B susceptibility of *Pseudomonas aeruginosa***. *Adv Microbiol* 2012, **2**(1).

61. Akasaka T, Tanaka M, Yamaguchi A, Sato K: **Type II topoisomerase mutations in fluoroquinolone-resistant clinical strains of *Pseudomonas aeruginosa* isolated in 1998 and 1999: role of target enzyme in mechanism of fluoroquinolone resistance**. *Antimicrobial agents and chemotherapy* 2001, **45**(8):2263-2268.

62. Lee JK, Lee YS, Park YK, Kim BS: **Alterations in the GyrA and GyrB subunits of topoisomerase II and the ParC and ParE subunits of topoisomerase IV in ciprofloxacin-resistant clinical isolates of *Pseudomonas aeruginosa***. *International journal of antimicrobial agents* 2005, **25**(4):290-295.

63. Rehman A, Jeukens J, Levesque RC, Lamont IL: **Gene-gene interactions dictate ciprofloxacin resistance in *Pseudomonas aeruginosa* and facilitate prediction of resistance phenotype from genome sequence data**. *Antimicrobial Agents and Chemotherapy* 2021.

64. Alvarez-Ortega C, Wiegand I, Olivares J, Hancock RE, Martinez JL: **Genetic determinants involved in the susceptibility of Pseudomonas aeruginosa to beta-lactam antibiotics**. *Antimicrob Agents Chemother* 2010, **54**(10):4159-4167.

65. Sun E, Gill EE, Falsafi R, Yeung A, Liu S, Hancock REW: **Broad-spectrum adaptive antibiotic resistance associated with *Pseudomonas aeruginosa* mucin-dependent surfing motility**. *Antimicrobial agents and chemotherapy* 2018, **62**(9).

66. Khaledi A, Weimann A, Schniederjans M, Asgari E, Kuo T-H, Oliver A, Cabot G, Kola A, Gastmeier P, Hogardt M *et al*: **Predicting antimicrobial resistance in *Pseudomonas aeruginosa* with machine learning-enabled molecular diagnostics**. *EMBO Mol Med* 2020, **12**(3):e10264-e10264.
